# Genome-wide analysis correlates *Ayurveda Prakriti*

**DOI:** 10.1038/srep15786

**Published:** 2015-10-29

**Authors:** Periyasamy Govindaraj, Sheikh Nizamuddin, Anugula Sharath, Vuskamalla Jyothi, Harish Rotti, Ritu Raval, Jayakrishna Nayak, Balakrishna K. Bhat, B. V. Prasanna, Pooja Shintre, Mayura Sule, Kalpana S. Joshi, Amrish P. Dedge, Ramachandra Bharadwaj, G. G. Gangadharan, Sreekumaran Nair, Puthiya M. Gopinath, Bhushan Patwardhan, Paturu Kondaiah, Kapaettu Satyamoorthy, Marthanda Varma Sankaran Valiathan, Kumarasamy Thangaraj

**Affiliations:** 1 CSIR-Centre for Cellular and Molecular Biology, Hyderabad, Telangana, India; 2 School of Life Sciences, Manipal University, Manipal, Karnataka, India; 3 Shri Dharmasthala Manjunatheshwara College of Ayurveda, Udupi, Karnataka, India; 4 Sinhgad College of Engineering, Pune, Maharashtra, India; 5 Foundation for Revitalization of Local Health Traditions, Bangalore, Karnataka, India; 6 Department of Statistics, Manipal University, Manipal, Karnataka, India; 7 Interdisciplinary School of Health Sciences, University of Pune, Pune, Maharashtra, India; 8 Department of Molecular Reproduction, Development and Genetics, Indian Institute of Science, Bangalore, Karnataka, India

## Abstract

The practice of *Ayurveda*, the traditional medicine of India, is based on the concept of three major constitutional types (Vata, Pitta and Kapha) defined as “*Prakriti*”. To the best of our knowledge, no study has convincingly correlated genomic variations with the classification of *Prakriti.* In the present study, we performed genome-wide SNP (single nucleotide polymorphism) analysis (Affymetrix, 6.0) of 262 well-classified male individuals (after screening 3416 subjects) belonging to three *Prakritis.* We found 52 SNPs (*p* ≤ 1 × 10^−5^) were significantly different between *Prakritis*, without any confounding effect of stratification, after 10^6^ permutations. Principal component analysis (PCA) of these SNPs classified 262 individuals into their respective groups (Vata, Pitta and Kapha) irrespective of their ancestry, which represent its power in categorization. We further validated our finding with 297 Indian population samples with known ancestry. Subsequently, we found that *PGM1* correlates with phenotype of Pitta as described in the ancient text of Caraka Samhita, suggesting that the phenotypic classification of India’s traditional medicine has a genetic basis; and its *Prakriti*-based practice in vogue for many centuries resonates with personalized medicine.

Among the traditional systems of medicine practiced all over the world, *Ayurveda* of India has a documented history dating back to 1500 BCE^1^,^2^. Though contemporary medicine is currently the mainstream of medical practice in India, *Ayurveda* is extensively used side by side and remains highly popular, especially in South Asia. The basic concepts of *Ayurveda* are; 1. five elements – panchabhuta – which constitute the physical universe including the human body and; 2. three doshas (Vata, Pitta and Kapha) or constitutional types of every human. These doshas refer broadly to the functions of motion, digestion and cumulation. Though all three doshas exist in every human being one is dominant based on which an individual’s *Prakriti* is determined. *Prakritis* are discreet phenotypes and they are determined on the basis of physical, psychological, physiological and behavioural traits, and independent of social, ethnic and geographical variables^1^,^3^,^4^. The etymology of these Sanskrit terms suggests that Vata originates from movement, Pitta from digestion and Kapha from cumulation. Since *Prakritis* underlie an individual’s predisposition to disease as well as response to treatment, it is imperative in *Ayurvedic* practice to identify the *Prakriti* of a patient before treatment^5^.

Concept of *Prakriti* in *Ayurveda* and its relationship with genomics was hypothesized over a decade ago^6^. Subsequent studies have attempted to correlate *Prakriti* classification with genetic information and association of single nucleotide polymorphisms (SNPs) in *HLA-DRB1*^7^, *CYP2C19*^8^, *EGLN1*^9^, inflammatory and oxidative stress related genes^10^, CD markers for various blood cells^11^,^12^, DNA methylation alterations^13^ and risk factors of cardiovascular or inflammatory diseases have been reported^14^. While these studies have shown the association of specific genes with the phenotype of a particular *Prakriti*, the association of genomic variations with *Prakriti* classification was lacking. This is the first attempt to classify the *Prakritis* using genome-wide SNP markers and to provide a scientific basis for *Prakriti* classification.

## Results and Discussions

**Figure 1:**
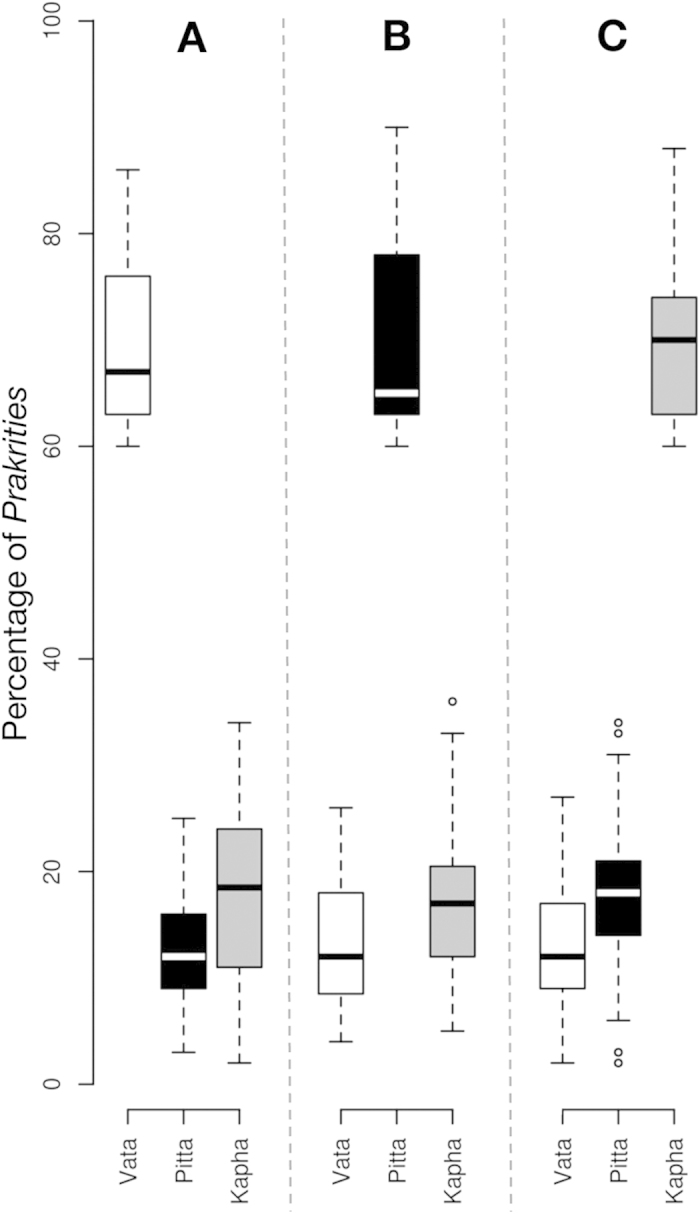
Box-plot representing the *Prakriti* proportion of subjects with Vata (94), Pitta (75) and Kapha (93) dominant characteristics. (**A**) Average percentage of Vata is 67%, while Pita and Kapha are 12% and 18.5%, respectively. (**B**) Average percentage of Pita is 65%, while Vata and Kapha are 12% and 17%, respectively. (**C**) Average percentage of Kapha is 70%, while Vata and Pita are 12% and 17%, respectively.

A total of 3,416 normal healthy male subjects between 20–30 years of age were recruited by the Institute of Ayurveda and Integrative Medicine (IAIM), Bangalore, Karnataka (‘B’ in tables); Sinhgad College of Engineering (SCE) Pune, Maharashtra (‘P’ in tables); and Shri Dharmasthala Manjunatheshwara College of Ayurveda (SDMCA), Udupi, Karnataka (‘U’ in tables). Since the hormonal fluctuations during premenstrual and menstrual phases result in numerous physical and psychological disturbances, which may have confounding effect at the time of *Prakriti* assessment, we have excluded females from this study (detailed justification on inclusion of only males is given in the Methods section). However, several studies have included of both male and female subjects for *Ayurveda*-based studies^7^,^8^,^15^,^16^. The subjects belonged to diverse ethnic and linguistic groups, and inhabited different geographical regions. The health status of every individual was ascertained by modern as well as *Ayurvedic* methods (details given in the Methods). The composition of *Prakriti* was determined by senior *Ayurvedic* physicians and confirmed independently by ‘AyuSoft’ (http://ayusoft.cdac.in), a software developed based on information from classical *Ayurvedic* literature. The subjects, whose *Prakriti* was in concordance between the assessment by *Ayurvedic* physicians and by AyuSoft were only selected for this study. Of the total 3,416 individuals evaluated, 971 had 60%–93% dominance of one *Prakriti* (Table S1), of which 262 individuals (94 Vata-dominant, 75 Pitta-dominant and 93 Kapha-dominant) with the highest proportion of one predominant *Prakriti* were randomly selected and subjected to genome-wide SNP analysis (Affymetrix array, 6.0) and genotypes were fetched using Birdsuite software^17^. The proportions of each dominant and co-dominant *Prakritis* are given in Fig. 1; Figure S1.

Out of 262 individuals analyzed, 245 passed the quality controls (QC) with the call rate 0.966 ± 0.0162 (Table S2). In order to validate the high-throughput data set, we randomly selected 48 markers from Affymetrix array and genotyped 48 individuals using custom-designed VeraCode GoldenGate Genotyping Assay System (Illumina, San Diego, USA). The call rate of VeraCode analysis was 99.61% and the genotype matched with Affymetrix data set (Table S3), suggesting that the genotypes obtained from Affymetrix array was genuine with minimum error (0.39%). Further, to increase the statistical power, we used Indian population data set as reference and imputation analysis was performed using Beagle (v3.3.1) software^18^ (Figure S1). As we had demonstrated earlier that Indian population has unique genetic architecture, we were skeptical of using non-Indian samples as a reference for imputation^19^. To evaluate our assumption, we masked 2%, 5% and 10% genotype of 207 unrelated Dravidian and Indo-European population samples and performed 110 simulations on chromosome 22 with four-reference populations *i.e.* Indian population (28 trios of Dravidian and Indo-Europeans; IN), different HapMap populations (CEU, YRI, CHB, CHS and JPT; HM), different South-Asian populations of 1000 genome project (BEB, GIH, ITU, PJL and STU; SA) and Indian along with HapMap populations (IH). As expected, imputed genotypes were more accurate with Indian samples (IN) [2% (0.9518 ± 0.0012); 5% (0.95045 ± 0.00109); 10% (0.9476 ± 0.0005)] compared to HM [2% (0.9462 ± 0.0013); 5% (0.9436 ± 0.0017); 10% (0.9396 ± 0.0005)], IH [2% (0.9463 ± 0.0014); 5% (0.9448 ± 0.0016); 10% (0.9417 ± 0.00066)] and SA [2% (0.9481 ± 0.0013); 5% (0.9471 ± 0.00098); 10% (0.9441 ± 0.00061)] samples (Table S4; Figure S2). In all the three masked data (2%, 5% and 10%), IN showed high imputation performance compared to HM, SA and IH. Even with ~10% masked data, the imputed genotypes were more accurate with IN than other references, suggesting that it is appropriate to use Indian data set for imputation. The data set of Gujarati Indians in Houston (GIH) is the only one available in the public domain, which was admixed recently, and hence does not truly represent the ANI-ASI ancestry of Indian population^19^,^20^. As the data were not suitable reference for imputation, we prepared our own reference panel of Indian population (http://www.ccmb.res.in/bic/database_pagelink.php?page=snpdata). To achieve this, we followed two steps (i) imputation of 15 trios of Indo-European and 15 trios of Dravidian, and (ii) imputation of 229 unrelated individuals imputed with the reference genotype obtained from step-I. Further, we used this reference for imputing the *Prakriti* individuals. In the first step, we found 10.5% and 17.8% Mendelian inconsistency in two trios, (Kashmiri Pandit) (Table S5), which were removed from the analysis. Finally, we obtained 791186 SNP markers with 0.95 ≤ R^2^ ≤ 1, for further analysis.

To make sure that the *Prakriti* samples were collected randomly and there was no major ancestral bias while collecting samples, we performed the principal component analysis (PCA)^21^ of 245 *Prakriti* samples (Figure S3). PCA analysis revealed no significant overall differences among the *Prakritis* (ANOVA p-value on eigenvector 1 V *vs.* K-0.434; V *vs.* P-0.89; P *vs.* K-0.51; and eigenvector 2 V *vs.* K-0.09; V *vs*. P-0.06; P *vs*. K-0.02). In order to check the ancestry of *Prakriti* individuals, we used our published data set of 297 Indian population samples with known ancestry^19^,^20^. These 297 samples include; 150 Dravidians, 80 Indo-European, 35 Austro-Asiatic, 27 Tibeto-Burman and 5 Great Andamanese (Table S6). We found 7,89,309 SNPs were common between *Prakriti* and Indian ancestral samples. In order to remove the differentiation on spurious axes^21^, we pruned 3,76,138 SNPs, which were in strong linkage disequilibrium (LD) (r^2^ > 0.75), and performed PCA with 4,13,171 SNPs. Our analysis showed that most of the *Prakriti* samples clustered with Dravidian and Indo-European (the two major ancestral population of India), and only 3 samples seemed to be Tibeto-Burman and admixed recently (Figure S4). Previous studies have shown that stratification could cause spurious association^22^,^23^,^24^,^25^, hence, PCA was performed^21^ using 4,05,782 SNPs (3,85,404 SNPs were pruned with r^2^ > 0.75) for 245 *Prakriti* samples, of which 40 were outliers and have been removed in 10 iterations with σ ≥ 6 on eigenvector 1 to 10 (Table S7; Figure S3 and S5). ANOVA analysis revealed that the *Prakriti* groups were not significantly different (p-value: V *vs.* P - 0.40 ± 0.28; V *vs.* K - 0.51 ± 0.32 and P *vs.* K - 0.48 ± 0.29) (Table S8); and 205 *Prakriti* samples were used for further analysis (Figure S1).

Association analysis was performed using plink software^26^. Since the present study has no cases and controls (patients and healthy), we considered one *Prakriti* as case and the remaining two *Prakritis* as controls, and performed association analyses in three combinations: Vata *vs*. Kapha and Pitta (V *vs.* PK); Pitta *vs*. Kapha and Vata (P *vs.* VK); Kapha *vs*. Pitta and Vata (K *vs.* VP). Prior to association analysis, 3,890; 4,153 and 4,124, respectively, markers were removed from 791186 markers, which were not in Hardy-Weinberg equilibrium (HWE) *i.e.* p-value < 0.001 in controls of V *vs.* PK, P *vs.* VK and K *vs.* VP; respectively. The three combination association results were further used to identify the SNPs that were significant. Considering the fact that none of the samples represents 100% single *Prakriti*, we did not expect very low p-value in the association analysis. In this scenario, truly associated loci may co-exist with false positive markers and can be identified by permutation analysis. As expected, we observed that SNPs having approximately same p-value in the extreme tail of theoretical distribution failed to achieve 10^6^ permutations (Table 1). For example, rs2939743 having p-value 7.61 × 10^−5^ dropped at 142717^th^ permutation while rs10197747 having p-value 2.50 × 10^−5^ achieved 10^6^ permutations, which of course revealed that rs2939743 is false positive. Similarly, we found 52 true positive SNPs achieved 1 million simulations with theoretical p-value ≤ 1 × 10^−5^ (details are given in Table 1; Figure S6).

**Figure 2:**
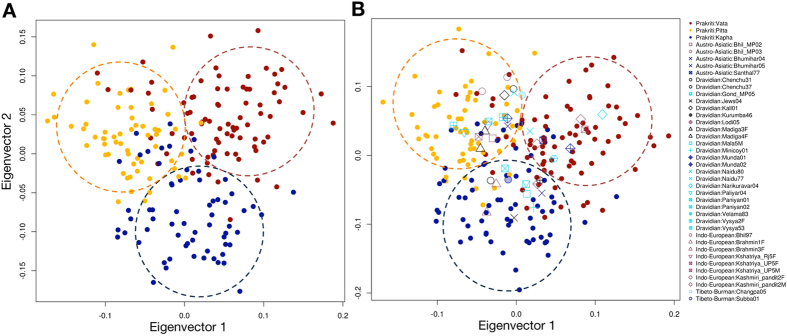
Principal component analysis (PCA) with 52 SNPs that showed p-value of <1 × 10-5 (**A**) PCA of Prakriti individuals showing three clusters (Vata, Pitta, Kapha), despite their linguistic, ethnic and geographical diversity. (**B**) PCA projection of Indian population samples with *Prakriti* individuals.

It is well known that some markers differ in allele frequency more across ancestral population, compared to other set of markers. Moreover, natural selection might be the reason for this phenomenon because it acts locus-specific manner^21^. We speculate that the above so-called true positive loci might be artifacts of population stratification because of high probability of false positive results at the p-value, which observed in association analysis. Hence, we performed extensive statistical analyses to control these confounding factors and/or population stratification. Prevailing methods include genomic control and EIGENSTRAT to find such confounding effect of stratification. Genomic control uses uniform inflation factor to correct stratification, which is not sufficient for those SNPs having high frequency differences between ancestors^21^. Hence, we proceeded with EIGENSTRAT and found p-value did not change drastically (Table S9). To further confirm, we used variance component model (implemented in EMMAX)^27^ and mixed-linear model of association analysis (implemented in GCTA)^28^, which can correct sample structure in association, but have different statistics comparative to eigenstrat. Intriguingly, even with this analysis, we did not observe any drastic change in the p-value (Table S9). This has proved that these 52 SNPs were genuine characteristics of *Prakriti* and not derived from ancestry. Moreover, we also explored the allele frequency differences between centers; however, we did not find any significant difference for these 52 SNPs (Table S10). We further explored the power of 52 SNPs in *Prakritis* genetic differentiation (Figure S1). In principal component analysis, 19 SNPs were excluded with r^2^ > 0.75 and, as expected, we found striking separation of subjects according to their *Prakriti* (Fig. 2A). On eigenvector-1 (eigenvalue = 18.168248) Pitta significantly differentiated against Vata and Kapha (p-value = 1.11022 × 10^−16^, 4.44089 × 10^−16^, respectively); while on eigenvector 2 (eigenvalue = 15.890861) Kapha was significantly different compared to Vata and Pitta (p-value = 3.33067 × 10^−16^ and ~0 respectively).

To examine the statistical power of these 52 markers for categorizing the samples with unknown *Prakriti*, we generated a statistical model (see methods). Initially, we applied it on 205 samples and found 23.9% (49 out of 205) were explained by the proposed model (Table S11). Further, we applied it on 297 Indian (population) samples and found 37 individuals (5 Austro-Asiatic; 22 Dravidian; 8 Indo-European and 2 Tibeto-Burman) satisfying the model. According to the model, 7 individuals were Vata, 20 were Pitta and 11 were Kapha. Interestingly, Indian population samples, which belong to one *Prakriti* were from different ancestry (Table S12), suggesting that these makers could separate the *Prakritis*, irrespective of their ancestry. To confirm the proposed model, we projected these 37 individuals on eigenvector of *Prakriti* samples and found that these individuals clustered with *Prakriti* as predicted in the model (Fig. 2B). It suggests that the cluster is based on *Prakriti*, and is not due to the ancestry of samples. That would also suggest that the phenotypic variations have a genetic basis, which would be shared by *Prakritis* of *Ayurveda*.

Further, we used these 52 markers to find the genotype-phenotype correlations. We observed that 2 markers (rs10518915 and rs986846) were associated with two different *Prakriti*; rs10518915 with Vata and Pitta, while rs986846 with Kapha and Vata. This observation prompted us to believe that different alleles of the same locus might be influencing different *Prakriti* (Table 1). In order to correlate the functional relevance of these SNPs, we divided them into genic and non-genic. The SNPs, which are within 10 kb of gene, were considered genic; while others as non-genic^29^,^30^. We found 28 were genic SNPs, of which 12 were in Vata (7 genes), 11 in Pitta (7 genes), and 6 in Kapha (7 genes) (Table 1). To correlate the function of these genes with respect to the characteristics of *Prakritis*, we searched in Kyoto Encyclopedia of Genes and Genomes (KEGG) pathway and Reactome event and found *PGM1* gene associated with the Pitta phenotype. In *Ayurveda*, characteristics of Pitta include digestion, metabolism and energy production. Interestingly, we found *PGM1* gene is in the center of many metabolic pathways *i.e.* glycolysis or gluconeogenesis (hsa00010); pentose phosphate pathway (hsa00030); galactose metabolism (hsa00052); purine metabolism (hsa00230) and; starch and sucrose metabolism (hsa00500) (Figure S7). Our finding suggests that the function of the gene directly correlates with the role of Pitta in metabolism as described in *Ayurvedic* literature.

In addition, we have checked the *PGM1* gene markers in Affymetrix data set and found 4 markers (rs2269241, rs2269240, rs2269239,and rs2269238) were associated with Pitta *Prakriti* and all are in strong Linkage Disequilibrium (LD) (Figure S8). Therefore, to find the functionally relevant variants, we sequenced the whole exons and UTRs of the *PGM1* gene in 78 individuals using Ion Torrent PGM (Life Technologies, USA). We found 23 variations in the gene, of which 8 were novel (Table S13). Interestingly, one non-synonymous; c.1258T > C (p.Tyr420His) (rs11208257) variant was present in the LD block and found in association with Pitta *Prakriti* (p-value–7.049 × 10^−3^). The frequency of the mutant allele “C” was 5.8% in Pitta and 20% in Kapha *Prakriti* (Table S14). This result prompted us to replicate the marker (rs11208257) in additional samples. We genotyped this marker (rs11208257) for 665 *Prakriti* individuals (299 Vata, 164 Pitta and 202 Kapha) using Sanger sequencing method. Initially, we analyzed the distribution of the genotype among participating centres and found “U” samples (collected from Udupi centre) were not in HWE (p-value - 0.04) (Table S15). Hence, we excluded 169 “U” samples from the analysis. Association analysis revealed that allelic and genotype distribution of the marker rs11208257 is significantly different in Pitta *Prakriti* against Vata and Kapha with p-value- 2.06 × 10^−2^; p-value- 6.16 × 10^−3^, respectively. Further, we explored the association between P *vs.* V and P *vs*. K; and found significant p-value - 7.61 × 10^−3^ and 2.35 × 10^−2^, respectively. The results would therefore suggest that Vata differs more from the Pitta *Prakriti* than Kapha (Table S16). We further screened 1108 randomly selected Indians and 992 HapMap samples and found that the frequency of mutant allele “C” was 17.9% among Indians, 15.5–17.6% in the Europeans, 14.5–18.8% in East Asians, 42% in Mexican, 15.3% in admixed Indians (GIH) and 12.8–28.3% in Africans. Indians have comparable frequency with Europeans and GIH (Table S17). Interestingly, we found Pitta has less frequency of mutant “C” allele, and Vata and Kapha have comparable frequency with overall Indian population. To explore the functional relevance of the variant, we used SIFT software and found that the mutation is damaging with 0.01 score and thus substitution at this position may affect the protein function. Our data suggest that the SNP (rs11208257) in *PGM1* gene is linked with one of the main features (energy production), which is more homogenous and constant in Pitta than with Vata and Kapha, and a genotype correlation exists for the characteristics of *Prakriti* classification.

In conclusion, our preliminary study suggests that the *Prakriti* classification, as a foundation for the practice of *Ayurveda*, has a genetic basis and does provide clues for further studies.

## Methods

### Selection of subjects and *Prakriti* assessment

Selection of subjects and evaluation of the *Prakriti* (the human classification of Indian ancient medicine) were carried out at three centres; 1. Institute of Ayurveda and Intergrative Medicine (IAIM), Bangalore, Karnataka; 2. Sinhgad College of Engineering (SCE) Pune, Maharashtra; and 3. Shri Dharmasthala Manjunatheshwara College of Ayurveda (SDMCA) Udupi, Karnataka. This study was approved by Institutional Ethics Committees (IECs) of all the collaborative centres and the methods were carried out in accordance with the approved guidelines. We have screened normal and healthy male subjects, who were between 20–30 years. Although several *Ayurveda*-based studies have included both male and female subjects^7^,^8^,^15^,^16^, we have excluded female subjects from this study to minimize the confounding variations. *Prakriti* of an individual is determined based on defined anatomical, physiological psychological and behavioural characteristics. During actual assessment of *Prakriti*, the *Ayurvedic* physician needs to factor in these characteristics. One such aspect is the cyclical hormonal changes that occur in women, particularly the menstrual cycle. The hormonal fluctuations result in numerous physical and psychological disturbances, which occur in the premenstrual and menstrual phases. Existing evidence suggest that about 97% of young nulliparous women experience varying degrees of such disturbances^31^. These elicitable and visible features can confound or obscure the *Prakriti* assessment process. For example, premenstrual irritability occurring in a woman of Kapha *Prakriti* is confounding, since Kapha *Prakriti* individuals normally possess low irritability. Although the *Ayurvedic* physicians routinely enquire about the menstrual habits of patients while assessing the *Prakriti*, it would have been difficult for us to make similar enquiries to young, healthy women who volunteered to join this study. The health status of an individual was assessed based on the *Ayurvedic* criteria, that include; normal desire for food, easy digestion of ingested food, excretion of feces, excretion of urine, excretion of flatus, functioning of sensory organs, comfortable sleep, easy awakening, and attainment of strength, bright complexion and longevity. Subjects with smoking habit, diabetes, hypertension and other chronic diseases were excluded from the study. Blood pressure (BP) was measured for each subject and BP > 130/90 mm of Hg were excluded from the study. Chronic systemic diseases such as rheumatoid arthritis, cancer, etc., and subjects having recent history of acute ailments such as fever due to infections were also excluded.

We followed three steps for the *Prakriti* assessment of each subjects. In the first stage, senior *Ayurvedic* physicians assessed the *Prakriti* of the subjects, applying classical *Ayurveda* parameters of *Prakriti* determination. In second stage, the same subjects were assessed using Ayusoft, a *Prakriti* software (www.ayusoft.cdac.in), which contains a comprehensive questionnaire, which had been developed based on the information from original *Ayurvedic* literature. In the third stage, another team of *Ayurvedic* physicians, who were not aware of the outcomes of assessment by senior physicians and Ayusoft, compared the *Prakritis* analysis. Subjects with ≥60% of single *Prakriti* dominance and having concordance in all the three stages were selected for the genome-wide analysis. Quantitative analysis of *Prakriti* was performed using Ayusoft along with traditional ayurvedic measures for the *Prakriti* assessment. The reason for considering ≥60% of a particular *Prakriti*as a dominant was mainly due to feasibility and concordance. Single dosha *Prakriti* with high percentage of one dosha rarely exist, hence most of the individuals possess dual-dosha *Prakriti*^12^. Therefore, we have considered subjects with ≥60% as single dosha dominant*Prakriti*. Subjects ≥60% of one dominant *Prakriti* were selected and blood was drawn after obtaining their informed written consent. A total of 3,416 healthy individuals were screened for their *Prakriti*, as per the details given above. From the total, 971 subjects who showed a predominant *Prakriti* of ≥60% were included in the analysis (Figure S1).

### High throughput genotyping, their quality control criteria and resequencing

#### Genotyping

DNA was isolated from the blood samples using standard protocol^32^. We randomly selected 262 *Prakriti* individuals for genotyping, using Genome-Wide Human SNP Array from Affymetrix (6.0), following manufacturer’s protocols. About 250 ng of genomic DNA was digested with *Nsp I* and *Sty I* restriction enzymes, followed by ligation of *Nsp*/*Sty*adaptors, using T4 DNA ligase. PCR was performed using the primers that are specific to these adopters. After checking the amplicons on 2% agarose gel, they were purified with deep-well plate using magnetic beads and the fragments were eluted using EB buffer, followed by quantification and fragmentation. The fragmented PCR products (<180 bp) were end-labeled using labeling kit. Labeled fragments were hybridized onto the Affymetrix (6.0) SNP arrays using hybridization cocktail. Hybridization was performed in hybridization oven for about 18 hrs at 50 °C. After hybridization, arrays were washed, stained, scanned and analyzed using Affymetrix Genotyping Console 2.0 and GeneChip® Operating Software (GCOS). The samples which passed the quality controls *i.e.* call rate >95% and CQC > 0.4 were considered. Affymetrix power tool (apt-geno-qc) was used for calculation of dm (dynamic model) value. The samples having dm.all_qc<0.83 were removed from further analysis and genotypes were fetched with Birdsuite software from Broad Institute^17^ (Figure S1).

#### Detection of technical artifacts

In order to validate the Affymetrix data set, we randomly selected 48 markers (Table S3) from Affymetrix array and genotyped 48 individuals, who were already genotyped by Affymetrix array, using custom-designed VeraCode GoldenGate Genotyping Assay System (Illumina, San Diego, USA). Genotyping was performed according to the manufacturer’s (Illumina, San Diego, USA) instructions. The genotypes obtained by both the platforms were compared and checked for accuracy (Figure S1).

#### Targeted resequencing

We sequenced the whole-exons and UTRs of *PGM1* gene (Figure S1) for randomly selected 43 Pitta and 35 Kapha individuals using Ion Torrent (Life Technologies, USA), following protocols of the manufacturer. Primer sequences were manufactured specifically for use with Ion AmpliSeq kits. The costume Ion AmpliSeq^TM^ primer contains 35 amplicons in a single pool. For preparing amplicon libraries, about 10 ng of DNA was amplified (PCR) using AmpliSeq^TM^ primer pools and Ion AmpliSeq^TM^ HiFi master mix (Ion AmpliSeq kit version 2.0 Beta). The amplified products were pooled and treated with 2 μl of FuPa reagent. The amplicons were then ligated with adapters from the Ion Xpress^TM^ barcoded adapters 17–64 kit according to the manufacturer’s instructions (Ion Torrent). After ligation, the amplicons were purified by Agencourt® AMPure® XP Reagent and additional amplification was performed to complete linkage between adapters and amplicons. In order to determine the library concentration, an Agilent 2100 Bioanalyzer high-sensitivity DNA kit (Agilent, Santa Clara, CA) was used to visualize the size range of the libraries. Equimolar concentrations of all the libraries were pooled and diluted. Using Ion One Touch^TM^ 200 Template Kit v@ DL (Life Technologies, USA), emulsion PCR was carried out according to the manufacturer’s instructions. Ion Spheres (ISPs) were recovered according to the Ion Sphere Particles 200 recovery protocol. Sequencing was done following the Ion PGM^TM^ 200 Sequencing Kit Protocol (version 6; Ion Torrent). The 318 sequencing chip was loaded and run on an Ion Torrent PGM (Ion Torrent). Base calling and alignment were performed using the Torrent Suite 3.0 software (Ion Torrent). In order to find the significant variation in the *PGM1* whole exome data, we performed association analysis using plink software^26^ and variations are annotated on EnsEMBL-BioMart.

#### Sanger sequencing

To validate and replicate the Pitta associated SNP (rs11208257), Sanger sequencing was carried out for 496 *Prakriti* samples (246 Vata, 116 Pitta and 134 Kapha) along with randomly selected 1108 Indian samples. Pair of primers (Forward primer: 5′- GCACGTTTCTTACAGCAGCT-3′ and Reverse primer: 5′-ACCTTACCTTGTACCCCAGC-3′) were designed, synthesized and PCR was performed on the GeneAmp 9700 Thermal Cycler (Applied Biosystems, Perkin-Elmer) using the following cycling conditions: 95 °C for 5 min, 35 cycles at 95 °C for 30 s, 58 °C for 30 s, 72 °C for 2 min and a final extension at 72 °C for 7 min. Amplicons were purified using with USB ExoSAP-IT (Affymetrix) according to the manufacturers instructions. The purified products were directly sequenced using the Big Dye Terminator cycle sequencing kit (Applied Biosystems, Foster City, CA, USA) and analyzed using 3730 DNA Analyzer (Applied Biosystems, Foster City, CA, USA)(Figure S1). The genotypes were noted, and statistical analysis was performed with plink^26^ and R.

### Indian, HapMap and 1000 genome project sample details

For comparative analysis, we used Affymetrix (6.0) array data of 297 well-classified Indian samples with known lingustic and ethnic affiliations *i.e.* 150 Dravidians, 80 Indo-European, 35 Austro-Asiatic, 27 Tibeto-Burman and 5 Great Andamanese. In addition, 15 trios of Dravidian (Vysya, Madiga, Mala; 5 each) and 15 trios of Indo-European (Kshatriya, Brahmin and Kashmiri Pandit; 5 each) were used for imputation^19^,^20^. We followed the same procedure (mentioned above) for extraction of genotypes and CQC measures. The list of population and their details are given in Table S6. We have also used 1184 HapMap (ftp://ftp.ncbi.nlm.nih.gov/hapmap/genotypes/2009-01_phaseIII/plink_format/) and 1000 genome project data (ftp://ftp.1000genomes.ebi.ac.uk/vol1/ftp/release/20130502/) for imputation and comparative analysis.

### Imputation and their relationship with ancestry

Imputation was performed for the missing genotypes using Beagle-v3.3.2 software^18^. In order to check the power of correct imputation, we randomly masked 2%, 5% and 10% genotype of chromosome 22 in 207 unrelated Indian population samples. We imputed masked genotypes with four types of reference *i.e.* Indian triose (15 trios of Dravidian and 15 trios of Indo-European), HapMap samples (CEU, YRI, CHB, CHS and JPT), Indian + HapMap samples and samples of 1000 genome project with South-Asian ancestry (BEB, GIH, ITU, PJL and STU). Accuracy of imputation was calculated by comparing imputed and true genotype in 110 simulations for 2%, 5% and 10% masked data. To perform the above analysis, we used a perl script.

To impute missing genotype in *Prakriti* samples, we followed two steps; In the first step, imputation and phasing was done for 15 trios of Dravidian (Vysya, Madiga, Mala; 5 trios each) and 15 trios of Indo-European (Kshatriya, Brahmin and Kashmiri Pandit; 5 trios each) together without reference population. SNPs which do not follow Mendelian rule in trios were checked and masked with Beagle utility program^18^. Number of SNPs per family, which do not follow Mendelian consistency are given in Table S5. In the second step, we performed imputation of unrelated population samples (Dravidian, Indo-European and Austro-Asiatic), with imputed familial (trios) samples as reference. Further, we imputed *Prakriti* samples with reference of imputed trios and unrelated Indian population samples and selected only those markers which were having R^2^ > 0.95 for further analysis.

### Population stratification

Principal component analysis was done with Eigensoft Package^21^. Convertf was used for converting plink ped file to Eigenstrat format. We pruned the SNPs on the basis of their Linkage Disequilibrium (r2 > 0.75) before running PCA by using Eigensoft’s killr^2^ option. Ten eigenvectors were fetched. To find the ancestry of 245 *Prakriti* samples, we used 297 known ancestry of Indian population dataset (previously published) and performed the PCA. Stratification was checked and 40 outlier samples were excluded with cutoff sigma value ≥0.6 (default value) on 1–10 eigenvectors in 10 iterations (Figure S1).

### Association analysis

Plink was used for association analysis^21^. Imputed Beagle file were converted into plink ped file. Association analysis was performed for the *Prakritis*. Since there are no case control groups in the present study, we compared one *Prakriti* against the other two Prakritis (Vata *vs.* Pitta and Kapha, Pitta *vs.* Vata and Kapha, and Kapha *vs.* Vata and Pitta) and calculated p-value from theoretical distribution. In order to exclude the markers, which could be in association by chance, we also performed adaptive permutation approach (empirical distribution) for maximum 10^6^ iteration withplink and considered only those markers who achieved maximum 10^6^ permutations and have p-value ≤ 1 × 10^−5^ in theoretical distribution (Figure S1).

### Addressing issue of population stratification as possible confounder in association analysis

Even subtle stratification can cause spurious association; hence we used EIGENSTRAT software^21^ for correcting association chi-square value on 10 eigenvector and to find its confounding effect. Initially we excluded 385, 404 SNPs with r2 > 0.75 and calculated eigenvector with remaining 405, 782 SNPs with SMARTPCA. Further we used these same 10 eigenvector for correction of chi-square value with EIGENSTRAT (Figure S1).

To address this issue, we also used EMMAX and GCTA tools^27^,^28^. Both statistical methods consider genetic structure in association analysis. Hence, we expected major changes in p-value of 52 SNPs. First, we generated IBS matrix implemented in EMMAX and then used it with 10 eigenvector (generated with SMARTPCA) as covariate to calculate p-value with variance component model (implemented in EMMAX). To calculate mixed-model association p-value (implemented in GCTA), first, we calculated genetic relationship matrix and 10 eigenvectors with GCTA; and used it in calculation of p-value (Figure S1).

### Statistical determination of *Prakriti* in subjects

In order to prove the power of these markers in samples of unknown *Prakriti* percentage, we generated a statistical model (Figure S1). First, this model applied to 205 Ayur samples and then replicated in Indian population data set with unknown *Prakriti*. For this, we calculated the weight for the genotype of each marker associated with the *Prakriti*. Suppose, if the frequency of genotype *g* in *Prakriti p* is *f*_*p*_ then the weight of *g*(W_gp_) can be calculated with equation (1)


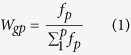


Un-standardized total weight of the *Prakriti* W_vs_ for a sample *s* with *n* number of associated markers for *p Prakriti* can be calculated using equation (2)


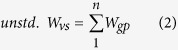


Hence, for a single sample there will be 3 weights W_vs_, W_ps_ and W_ks_ corresponding to Vata, Pitta and Kapha using equation (2). For making weights comparable, we standardized by subtracting with mean and dividing it by standard deviation. Mean and standard deviation were calculated from total weight of each sample for each markers corresponding to each *Prakriti*. If total number of sample is *N* then standardized weight can be calculated using equation (3)


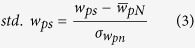


*Prakriti* is relative proposition (tridosha), so we calculated the differences of standardized weight for all 6 permutations; Δ_VP_, Δ_VK_, Δ_PV_, Δ_PK_, Δ_KP_ and Δ_KP_ for each samples and calculated representative statistics R_p_. For example, representative statistics for Kapha R_k_ can be calculated using equation (4)


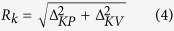


Since multiplication of 2 negative values is positive, the R_p_ value could be positive for 2 negative Δ values. Hence, we considered only those R_p_ values, which have both Δ value positive. Moreover, we consider only those samples which have R_p_ ≥ 3 to find dominant *Prakriti*. We applied this model to Indian population and selected 37 samples on the basis of R_p_ and Δ values.

### Phenotype and genotype correlation

We considered markers within 10 kb flanking region of gene as genic and other as non-genic. Physical location of the genes (knownGene.txt.gz) and SNPs (snp135.txt.gz) were fetched from http://hgdownload.soe.ucsc.edu/goldenPath/hg19/database/ and; windowbed(Bedtools: https://code.google.com/p/bedtools/) was used to find the SNPs within 10 kb flanking region of the genes. Only genic markers were used for genotype-phenotype correlation. Genic SNPs were selected and considered for further analysis. To correlate the function of associated markers with characteristics of the individual *Prakriti*, we checked in KEGG (Kyoto Encyclopedia of Genes and Genomes) pathway and Reactome event using NCBI2R R package.We used SIFT algorithm (http://sift.jcvi.org) for predicting the effects of non-synonomous variant (rs11208257) on protein function.

## Additional Information

**How to cite this article**: Govindaraj, P. *et al*. Genome-wide analysis correlates *Ayurveda Prakriti*. *Sci. Rep.* **5**, 15786; doi: 10.1038/srep15786 (2015).

## Supplementary Material

Supplementary Information

## Figures and Tables

**Table 1:** Empirical and theoretical distribution of the associated markers (p-value ≤1 × 10^−5^) of V*vs.* PK, P*vs.*VK and K*vs.*VP.

Chr	rsIDs	Gene	Position(hg19)	Minorallele	Theoretical distribution						Empirical distribution		
					MAFPrakriti I	MAFPrakritiII + III	Majorallele	Chi-square	P-value	ODD-ratio	P-value	No.of permutation	Passed inpermutation
**Vata**													
2	rs10197747	*–*	57644785	C	0.06875	0	A	17.66	2.64E*–*05	NA	2.50E*–*05	1000000	Passed
3	rs10446349	*VPS8*	186131720	G	0.6	0.4	A	15.64	7.66E*–*05	2.25	2.40E*–*05	1000000	Passed
1	rs10489167	*NFYC*	40949053	A	0.15	0.036	G	17.13	3.49E*–*05	4.725	6.57E*–*05	639360	–
15	rs10518915	*CGNL1*	55472117	C	0.6062	0.372	A	21.53	3.49E*–*06	2.599	4.00E*–*06	1000000	Passed
5	rs10866665	*–*	172908297	G	0.325	0.54	A	18.18	2.01E*–*05	0.4102	3.90E*–*05	1000000	Passed
10	rs10882293	*PDE6C*	95401687	T	0.6188	0.416	C	16.04	6.19E*–*05	2.278	6.60E*–*05	636363	–
5	rs11134808	*–*	172909959	G	0.325	0.536	A	17.52	2.84E*–*05	0.4168	4.55E*–*05	924075	–
7	rs11768155	*SEMA3C*	80203792	C	0.1062	0.016	T	16.35	5.26E*–*05	7.311	8.56E*–*05	490509	–
10	rs11815547	*–*	13647633	T	0.1688	0.044	A	18.06	2.15E*–*05	4.411	8.19E*–*05	513000	–
2	rs11902715	*FIGN*	164190114	G	0.2062	0.068	A	17.41	3.01E*–*05	3.561	3.60E*–*05	1000000	Passed
10	rs12266365	*PDE6C*	95398869	C	0.6188	0.416	A	16.04	6.19E*–*05	2.278	6.60E*–*05	636363	–
2	rs12473563	*–*	226605892	G	0.2375	0.452	A	19.29	1.12E*–*05	0.3776	6.48E*–*05	648351	–
6	rs12526892	*PACRG,PACRG–AS1*	163641063	A	0.15	0.344	G	18.7	1.53E*–*05	0.3365	6.00E*–*06	1000000	Passed
10	rs12781996	*–*	108025031	A	0.1	0.26	G	15.75	7.21E*–*05	0.3162	3.10E*–*05	1000000	Passed
2	rs13006806	*–*	226606872	C	0.2375	0.452	G	19.29	1.12E*–*05	0.3776	6.48E*–*05	648351	–
10	rs1326217	*PDE6C*	95400989	T	0.6188	0.404	A	18.01	2.20E*–*05	2.394	3.10E*–*05	1000000	Passed
8	rs13273017	*RIMS2*	104994931	T	0.4875	0.288	C	16.74	4.30E*–*05	2.352	7.83E*–*05	549450	–
2	rs13396046	*–*	57707740	A	0.06875	0	G	17.66	2.64E*–*05	NA	2.50E*–*05	1000000	Passed
2	rs1376616	*–*	79479938	C	0.1	0.012	T	17.1	3.56E*–*05	9.148	3.60E*–*05	1000000	Passed
11	rs1439528	*–*	96115783	A	0.625	0.408	G	18.38	1.81E*–*05	2.418	1.20E*–*05	1000000	Passed
2	rs1440472	*FIGN*	164193843	T	0.2625	0.108	C	16.64	4.52E*–*05	2.94	2.60E*–*05	1000000	Passed
15	rs1664454	*CGNL1*	55469925	T	0.5188	0.288	C	22.09	2.60E*–*06	2.665	5.00E*–*06	1000000	Passed
8	rs1845928	*RIMS2*	104977275	C	0.4875	0.288	T	16.74	4.30E*–*05	2.352	7.83E*–*05	549450	–
7	rs1949971	*SEMA3C*	80212278	A	0.1062	0.016	G	16.35	5.26E*–*05	7.311	8.56E*–*05	490509	–
1	rs2744803	*NFYC*	40955692	T	0.15	0.036	C	17.13	3.49E*–*05	4.725	6.57E*–*05	639360	–
21	rs2825953	*–*	20315181	A	0.3312	0.152	G	18.15	2.04E*–*05	2.763	5.67E*–*05	741000	–
10	rs2901714	*PDE6C*	95399009	A	0.6125	0.4	T	17.64	2.67E*–*05	2.371	2.50E*–*05	1000000	Passed
4	rs2939743	*C4orf19*	37219957	A	0.5375	0.34	G	15.65	7.61E*–*05	2.256	0.0002943	142714	–
12	rs3759173	*TBX3*	113607258	A	0.15	0.032	C	18.88	1.39E*–*05	5.338	2.30E*–*05	1000000	Passed
7	rs41486545	*SEMA3C*	80216538	G	0.1062	0.016	A	16.35	5.26E*–*05	7.311	8.56E*–*05	490509	–
8	rs4463386	*RIMS2*	104955665	A	0.4813	0.288	C	15.75	7.23E*–*05	2.294	7.83E*–*05	549450	–
8	rs4467913	*RIMS2*	105104591	A	0.4437	0.252	G	16.3	5.39E*–*05	2.368	5.46E*–*05	770000	–
16	rs4843892	*–*	84627121	A	0.6	0.4	G	15.64	7.66E*–*05	2.25	7.99E*–*05	525474	–
3	rs6443999	*VPS8*	186056249	C	0.6062	0.404	T	15.98	6.40E*–*05	2.271	5.96E*–*05	705294	–
10	rs6583901	*PDE6C*	95399988	T	0.3438	0.152	C	20.45	6.11E*–*06	2.922	3.30E*–*05	1000000	Passed
1	rs6586471	*–*	104648021	T	0.5125	0.292	C	20.17	7.07E*–*06	2.549	4.00E*–*06	1000000	Passed
2	rs6731290	*FIGN*	164196489	C	0.1812	0.056	T	16.3	5.40E*–*05	3.732	3.70E*–*05	1000000	Passed
2	rs6748662	*–*	57710603	G	0.06875	0	A	17.66	2.64E*–*05	NA	2.50E*–*05	1000000	Passed
5	rs7447285	*LOC643201*	175541987	T	0.075	0.004	C	16.02	6.27E*–*05	20.19	5.79E*–*05	726000	*–*
5	rs7717957	*–*	172914029	C	0.325	0.54	G	18.18	2.01E*–*05	0.4102	3.70E*–*05	1000000	Passed
1	rs823681	*NFYC,MIR30C1*	41004011	T	0.15	0.036	C	17.13	3.49E*–*05	4.725	6.57E*–*05	639360	–
1	rs9438945	*RIMS3*	40912171	C	0.15	0.036	G	17.13	3.49E*–*05	4.725	6.57E*–*05	639360	–
13	rs9547240	*LINC00351*	84973191	A	0.1875	0.056	G	17.61	2.71E*–*05	3.89	6.75E*–*05	622000	–
3	rs9815900	*–*	45321192	G	0.4062	0.22	C	16.35	5.27E*–*05	2.426	8.03E*–*05	523000	–
3	rs9830734	*VPS8*	186033195	T	0.625	0.408	C	18.38	1.81E*–*05	2.418	5.00E*–*06	1000000	Passed
2	rs986846	*–*	239172751	T	0.5813	0.36	C	19.33	1.10E*–*05	2.468	1.50E*–*05	1000000	Passed
**Pitta**													
12	rs1005375	*–*	3307020	T	0.6083	0.3862	C	16.91	3.91E*–*05	2.468	9.18E*–*05	457542	–
16	rs10163360	*RBFOX1*	6622152	A	0.2833	0.1172	G	16.92	3.89E*–*05	2.977	7.79E*–*05	539460	–
2	rs10192833	*SLC8A1*	40480999	C	0.3417	0.1621	T	16.24	5.58E*–*05	2.683	6.52E*–*05	644000	–
7	rs10231685	*LHFPL3*	103963525	G	0.3	0.1207	A	19.06	1.27E*–*05	3.122	2.00E*–*05	1000000	Passed
15	rs10518915	*CGNL1*	55472117	C	0.3	0.531	A	18.22	1.97E*–*05	0.3785	2.90E*–*05	1000000	Passed
10	rs10994942	*–*	63260442	A	0.525	0.3103	G	16.72	4.33E*–*05	2.456	6.05E*–*05	694305	–
10	rs10994948	*–*	63270054	G	0.5	0.2793	A	18.32	1.87E*–*05	2.58	8.00E*–*06	1000000	Passed
9	rs11792644	*–*	121598411	T	0.09167	0.01034	A	17.02	3.70E*–*05	9.654	9.39E*–*05	447104	–
16	rs12373036	*–*	54058839	A	0.4	0.2069	G	16.31	5.37E*–*05	2.556	0.000101	416000	–
3	rs12490846	*PTPRG*	61700707	A	0.2667	0.09655	G	19.66	9.24E*–*06	3.403	8.71E*–*05	482000	–
13	rs12584835	*–*	104098930	A	0.325	0.1483	G	16.57	4.69E*–*05	2.766	8.04E*–*05	522477	–
7	rs13233643	*LHFPL3*	103961859	T	0.2917	0.1207	C	17.53	2.84E*–*05	3	6.12E*–*05	686313	–
2	rs13423251	*NCKAP5*	133952350	A	0.3	0.131	G	16.38	5.18E*–*05	2.842	0.0001232	341000	–
16	rs1558564	*RBFOX1*	6620427	A	0.35	0.1448	T	21.93	2.82E*–*06	3.179	4.45E*–*05	944000	–
3	rs1602754	*–*	104529406	C	0.1167	0.01724	T	18.99	1.32E*–*05	7.528	6.79E*–*05	619000	–
6	rs16890791	*LCA5*	80244701	C	0.375	0.1828	T	17.25	3.29E*–*05	2.683	9.71E*–*05	432567	–
3	rs17065308	*PTPRG*	61722777	C	0.375	0.1862	T	16.52	4.82E*–*05	2.622	7.96E*–*05	527472	–
14	rs17124831	*C14orf166*	51523799	C	0.4583	0.2483	T	17.52	2.85E*–*05	2.562	7.83E*–*05	536463	–
13	rs2209540	*–*	94437798	G	0.2583	0.09655	T	18.03	2.17E*–*05	3.259	5.36E*–*05	783000	–
1	rs2269238	*PGM1*	63890125	A	0.075	0.2552	C	17.07	3.61E-05	0.2367	3.30E*–*05	1000000	Passed
1	rs2269239	*PGM1*	63881947	C	0.075	0.2586	G	17.57	2.77E*–*05	0.2324	1.90E*–*05	1000000	Passed
1	rs2269240	*PGM1*	63881852	G	0.075	0.2586	A	17.57	2.77E*–*05	0.2324	1.90E*–*05	1000000	Passed
1	rs2269241	*PGM1*	63881359	G	0.075	0.2621	A	18.07	2.12E*–*05	0.2283	2.20E*–*05	1000000	Passed
15	rs2388017	*–*	91094840	T	0.2917	0.5276	C	19.03	1.29E*–*05	0.3687	3.00E*–*05	1000000	Passed
10	rs2398215	*–*	10425666	A	0.2667	0.4897	G	17.28	3.23E*–*05	0.379	7.00E*–*06	1000000	Passed
6	rs2655668	*LCA5*	80248839	C	0.45	0.2345	T	18.86	1.41E*–*05	2.671	1.90E*–*05	1000000	Passed
6	rs2655670	*LCA5*	80251062	A	0.3917	0.1966	G	17.07	3.61E*–*05	2.632	5.81E*–*05	723000	–
6	rs2655688	*–*	80316792	G	0.5917	0.3724	C	16.59	4.65E*–*05	2.442	8.02E*–*05	524000	–
21	rs2835262	*SETD4*	36349861	C	0.1333	0.02759	T	17.22	3.32E*–*05	5.423	0.0001224	343000	–
15	rs2892383	*–*	91093506	T	0.3	0.5414	C	19.85	8.38E*–*06	0.3631	2.00E*–*05	1000000	Passed
5	rs420150	*–*	141164192	A	0.275	0.1034	C	19.21	1.17E*–*05	3.287	1.30E*–*05	1000000	Passed
3	rs4688663	*PTPRG*	61716091	C	0.3667	0.1793	T	16.61	4.58E*–*05	2.65	8.08E*–*05	520000	–
17	rs4789092	*RAB37,CD300LF*	70203226	C	0.1917	0.05517	A	18.37	1.82E*–*05	4.061	1.50E*–*05	1000000	Passed
1	rs619413	*HSD52*	59394979	T	0.2583	0.4862	G	18.1	2.10E*–*05	0.3681	1.90E*–*05	1000000	Passed
2	rs6546604	*–*	70496311	A	0.4	0.2	G	17.71	2.57E*–*05	2.667	6.08E*–*05	691308	–
2	rs6719806	*SLC8A1*	40512777	C	0.35	0.1655	A	16.86	4.02E*–*05	2.715	5.05E*–*05	831000	–
7	rs6969323	*LHFPL3*	104001723	A	0.3333	0.1483	C	18	2.21E*–*05	2.872	3.50E*–*05	1000000	Passed
15	rs7162785	*–*	91094879	A	0.3167	0.5379	C	16.67	4.45E*–*05	0.3981	8.28E*–*05	507492	–
16	rs7200031	*RBFOX1*	6606974	C	0.3083	0.1345	A	16.99	3.76E*–*05	2.869	6.77E*–*05	620000	–
17	rs739354	*GAS7*	9945013	C	0.1917	0.05862	A	17.06	3.61E*–*05	3.808	8.59E*–*05	489000	–
6	rs767789	*LCA5*	80290087	T	0.5917	0.3655	C	17.69	2.59E*–*05	2.515	3.70E*–*05	1000000	Passed
6	rs790607	*–*	91163700	A	0.3083	0.5276	G	16.41	5.11E*–*05	0.3992	3.70E*–*05	1000000	Passed
14	rs8007410	*–*	55637508	G	0.375	0.1862	T	16.52	4.82E*–*05	2.622	8.49E*–*05	494505	–
13	rs9540498	*–*	33802831	C	0.375	0.1793	G	18	2.21E*–*05	2.746	7.41E*–*05	567000	–
**Kapha**													
8	rs10086758	*–*	76924738	T	0.1	0.2929	G	18.55	1.66E*–*05	0.2683	9.28E*–*05	452547	–
9	rs10114425	*–*	110192021	T	0.1846	0.05357	C	17.71	2.57E*–*05	4	0.000112	375000	–
12	rs10778435	*CASC18*	104643081	A	0.3077	0.1393	G	16.19	5.74E*–*05	2.746	8.72E*–*05	481518	–
12	rs10848194	*–*	129791290	A	0.2615	0.4893	C	18.94	1.35E*–*05	0.3697	1.80E*–*05	1000000	Passed
12	rs10861491	*CASC18*	104643051	G	0.3077	0.1393	A	16.19	5.74E*–*05	2.746	8.72E*–*05	481518	–
1	rs11166345	*–*	100045548	A	0.2846	0.5107	G	18.43	1.76E*–*05	0.3812	1.70E*–*05	1000000	Passed
3	rs11714306	*CLSTN2*	141227150	C	0.6308	0.4143	T	16.66	4.47E*–*05	2.415	4.94E*–*05	850149	–
4	rs11933940	*C4orf19*	37238765	A	0.2769	0.4893	G	16.42	5.09E*–*05	0.3998	0.0001007	417000	–
2	rs12473563	*–*	226605892	G	0.5154	0.3	A	17.7	2.58E*–*05	2.481	8.75E*–*05	480000	–
2	rs13006806	*–*	226606872	C	0.5154	0.3	G	17.7	2.58E*–*05	2.481	8.75E*–*05	480000	–
2	rs13429987	*–*	184201539	G	0.1308	0.3179	A	16.21	5.67E*–*05	0.3229	3.90E*–*05	1000000	Passed
2	rs1438266	*KYNU*	143363789	A	0.4692	0.2643	G	16.88	3.97E*–*05	2.461	8.58E*–*05	489510	–
5	rs152270	*–*	141123895	C	0.02308	0.1571	T	15.72	7.34E*–*05	0.1267	8.82E*–*05	476000	–
5	rs1553239	*–*	30841081	T	0.2077	0.06786	C	17.43	2.98E*–*05	3.601	5.78E*–*05	726273	–
5	rs17592250	*LOC101929380*	86526909	T	0.09231	0.01071	C	16.77	4.22E*–*05	9.39	8.43E*–*05	498501	–
10	rs17731	*KLF6*	3811561	A	0.4846	0.2464	G	23.07	1.56E*–*06	2.875	2.00E*–*06	1000000	Passed
18	rs17804058	*CD226*	65755221	G	0.1615	0.03929	C	18.44	1.75E*–*05	4.711	8.72E*–*05	481518	–
14	rs1862127	*ADCK1*	77477049	T	0.3385	0.15	C	19.02	1.29E*–*05	2.899	2.00E*–*06	1000000	Passed
14	rs2105269	*GALNT16*	68816987	T	0.6385	0.4214	C	16.73	4.30E*–*05	2.424	6.67E*–*05	629370	–
1	rs213490	*SSBP3*	54627572	T	0.5769	0.35	C	18.74	1.50E*–*05	2.532	1.90E*–*05	1000000	Passed
20	rs2327233	*–*	9808517	C	0.5462	0.3393	T	15.77	7.16E*–*05	2.343	5.45E*–*05	771000	–
20	rs2876175	*–*	9820828	C	0.3462	0.5607	T	16.35	5.26E*–*05	0.4148	4.21E*–*05	998000	–
4	rs2939743	*C4orf19*	37219957	A	0.2462	0.4964	G	22.87	1.73E*–*06	0.3312	5.00E*–*06	1000000	Passed
12	rs3751176	*–*	127241860	A	0.1846	0.3821	G	15.93	6.57E*–*05	0.3661	7.84E*–*05	536000	–
14	rs4020132	*–*	77481947	A	0.3385	0.15	G	19.02	1.29E*–*05	2.899	2.00E*–*06	1000000	Passed
4	rs4107575	*C4orf19*	37238678	A	0.2769	0.4893	G	16.42	5.09E*–*05	0.3998	0.0001007	417000	–
14	rs4903671	*–*	77481783	A	0.3231	0.1357	C	19.84	8.40E*–*06	3.039	6.00E*–*06	1000000	Passed
22	rs5765425	*FBLN1*	44271097	A	0.3462	0.5607	G	16.35	5.26E*–*05	0.4148	4.82E*–*05	872127	–
20	rs6039614	*–*	9815136	C	0.3462	0.5643	A	16.9	3.94E*–*05	0.4088	3.30E*–*05	1000000	Passed
20	rs6071392	*–*	58880119	T	0.3231	0.15	C	16.33	5.33E*–*05	2.705	4.21E*–*05	998000	–
20	rs6118782	*–*	9811911	C	0.3538	0.5679	T	16.26	5.51E*–*05	0.4167	5.02E*–*05	837162	–
1	rs6688707	*–*	100040996	T	0.2846	0.5107	G	18.43	1.76E*–*05	0.3812	1.70E*–*05	1000000	Passed
2	rs6733573	*–*	204840871	T	0.3308	0.1536	C	16.82	4.12E*–*05	2.724	5.29E*–*05	794000	–
3	rs6770415	*–*	13242771	G	0.3231	0.1429	A	18.02	2.18E*–*05	2.864	7.16E*–*05	587000	–
3	rs7616568	*–*	2045846	G	0.3385	0.5643	A	18.11	2.08E*–*05	0.3951	1.20E*–*05	1000000	Passed
8	rs7845105	*TRAPPC9*	140978153	T	0.06154	0	G	17.57	2.76E*–*05	NA	6.90E*–*05	609000	–
15	rs8026848	*FAM189A1*	27361149	C	0.2231	0.07857	G	17.02	3.70E*–*05	3.367	5.58E*–*05	753000	–
19	rs8103262	*ZNF808,ZNF701*	57757626	C	0.3	0.125	T	18.38	1.81E*–*05	3	3.30E*–*05	1000000	Passed
6	rs9466669	*–*	23297836	A	0.06154	0	G	17.57	2.76E*–*05	NA	8.16E*–*05	514485	–
3	rs9815240	*PLSCR4*	147394867	C	0.1538	0.03214	A	20.01	7.72E*–*06	5.475	1.70E*–*05	1000000	Passed
2	rs986846	*–*	239172751	T	0.3	0.5143	C	16.5	4.87E*–*05	0.4048	2.40E*–*05	1000000	Passed
