# Supplementary material for: Genome-wide analysis correlates Ayurveda Prakriti
Source: Sci Rep. 2015 Oct 29;5:15786. doi: 10.1038/srep15786 (PMC4625161; doi:10.1038/srep15786)
Supplement: Supplementary Information [file srep15786-s1.pdf]

Supplementary materials

Genome-wide analysis correlates *Ayurveda Prakriti*

Periyasamy Govindaraj[1,+], Sheikh Nizamuddin[1,+], Anugula Sharath[1], Vuskamalla Jyothi[1], Harish Rotti[2], Ritu Raval[2], Jayakrishna Nayak[3], Balakrishna K Bhat[3], B.V. Prasanna[3], Pooja Shintre[4], Mayura Sule[4], Kalpana S. Joshi[4], Amrish P. Dedge[4], Ramachandra Bharadwaj[5], G.G. Gangadharan[5], Sreekumaran Nair[6], Puthiya M Gopinath[2], Bhushan Patwardhan[7], Paturu Kondaiah[8], Kapaettu Satyamoorthy[2], Marthanda Varma Sankaran Valiathan[2], Kumarasamy Thangaraj[1,*]

[1]CSIR-Centre for Cellular and Molecular Biology, Hyderabad, Telangana, India
[2]School of Life Sciences, Manipal University, Manipal, Karnataka, India,
[3]Shri Dharmasthala Manjunatheshwara College of Ayurveda, Udupi, Karnataka, India
[4]Sinhgad College of Engineering, Pune, Maharashtra, India
[5]Foundation for Revitalization of Local Health Traditions, Bangalore, Karnataka, India
[6]Department of Statistics, Manipal University, Manipal, Karnataka, India,
[7]Interdisciplinary School of Health Sciences, University of Pune, Pune, Maharashtra, India
[8]Department of Molecular Reproduction, Development and Genetics, Indian Institute of Science, Bangalore, Karnataka, India

[+]These authors contributed equally to this work.

[*]Corresponds to:

Kumarasamy Thangaraj
CSIR-Centre for Cellular and Molecular Biology
Hyderabad 500 007, India
Phone: +91-40-27192828
Fax: +91-40-27160591
E-mail:thangs@ccmb.res.in

Supplementary Figures

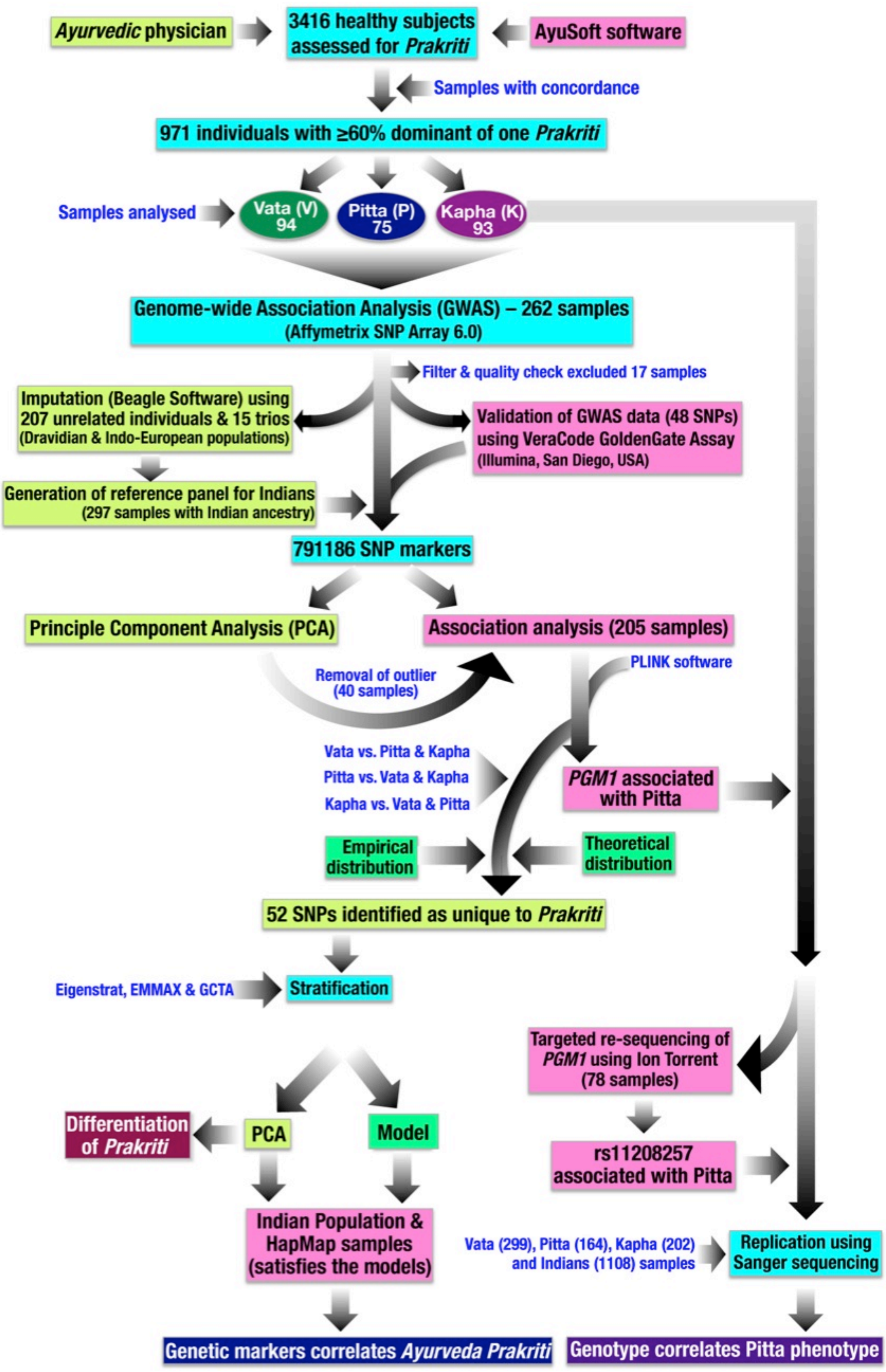

Figure S1. Schematic representation of methodologies/workflow used in this study.

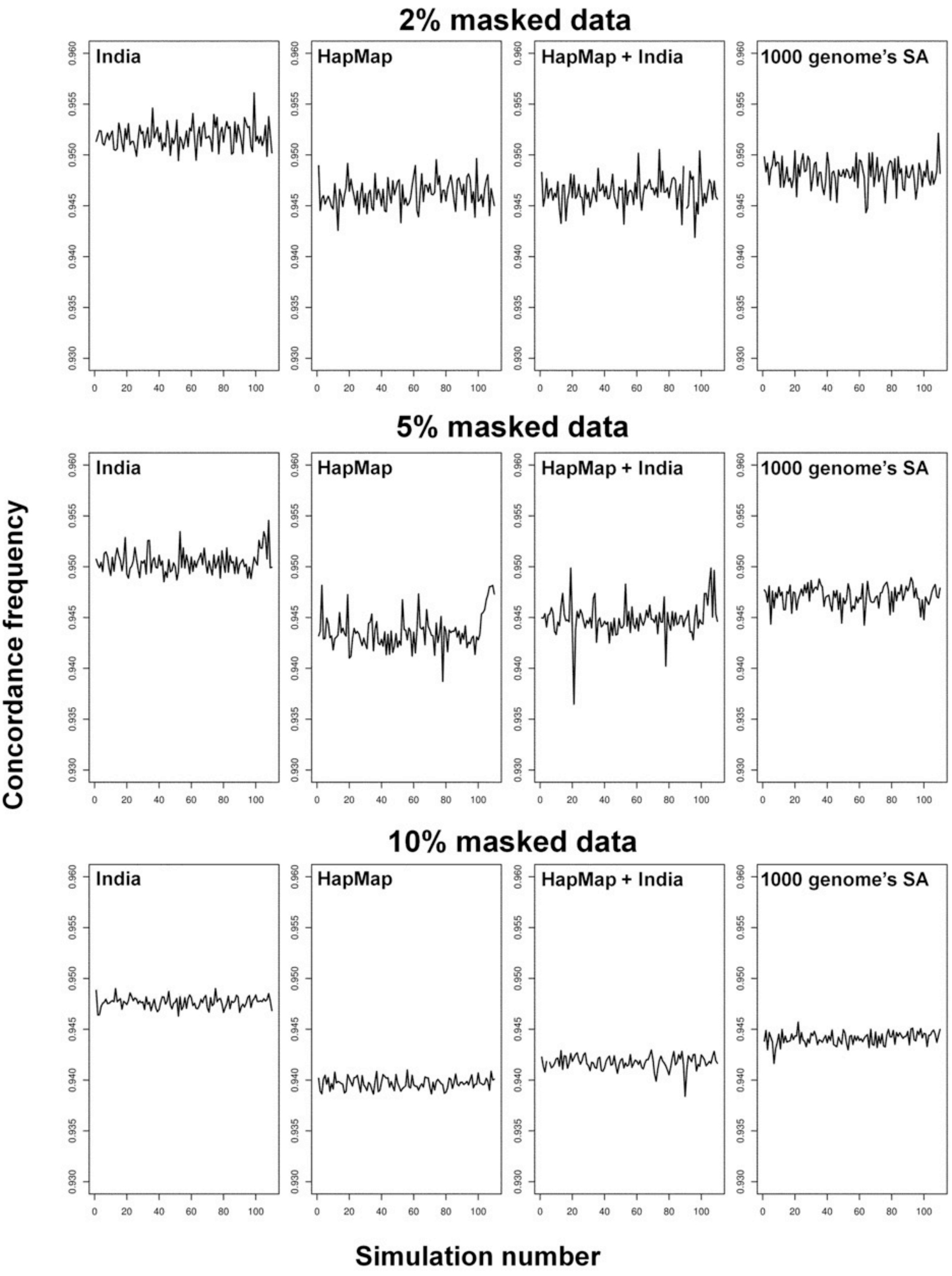

Figure S2. Genotype imputation and their performance on 2%, 5% and 10% masked data with different reference population (India, HapMap, India+HapMap and 1000 Genome project's South-Asian samples).

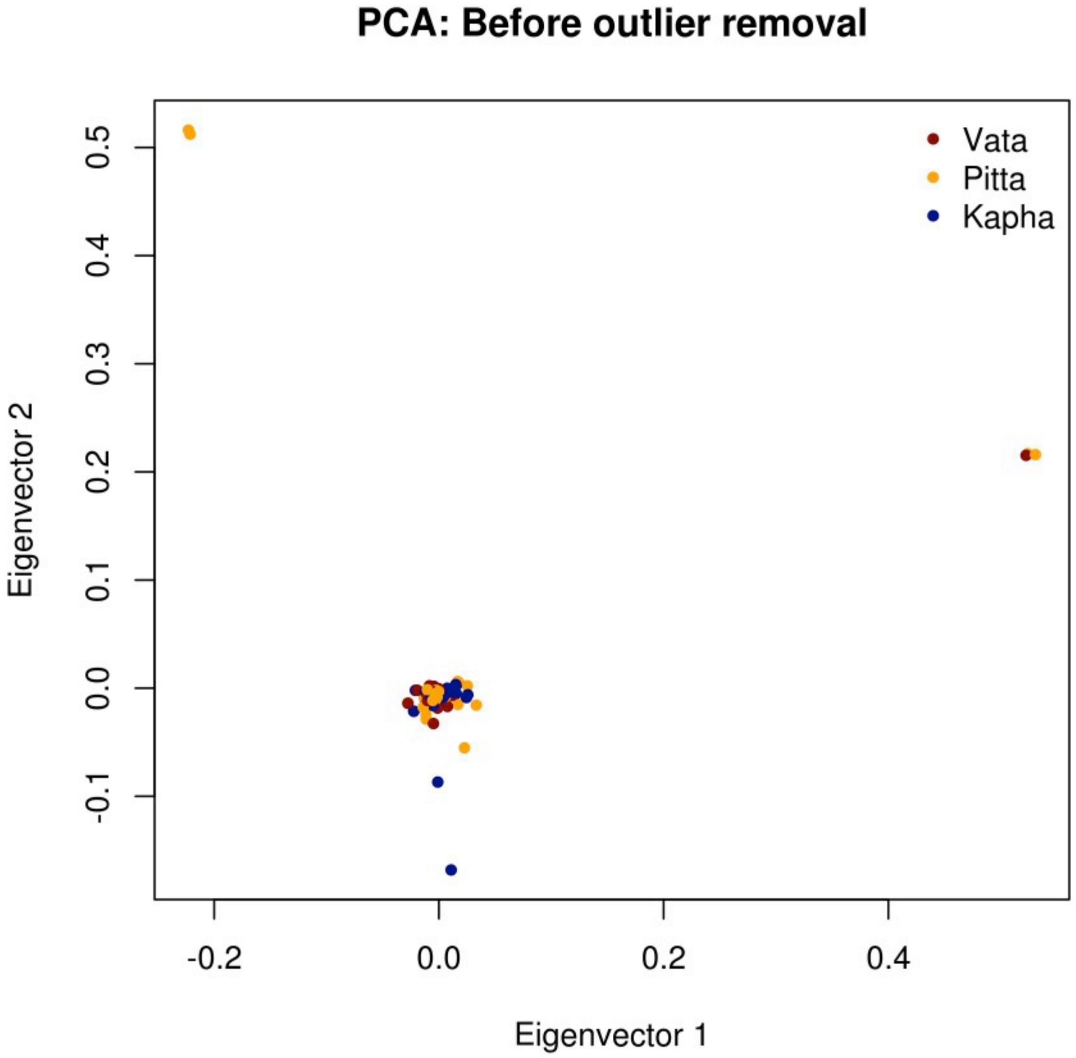

Figure S3. Principal component analysis (PCA) of 245 *Prakriti* individuals using 405,782 SNP markers.

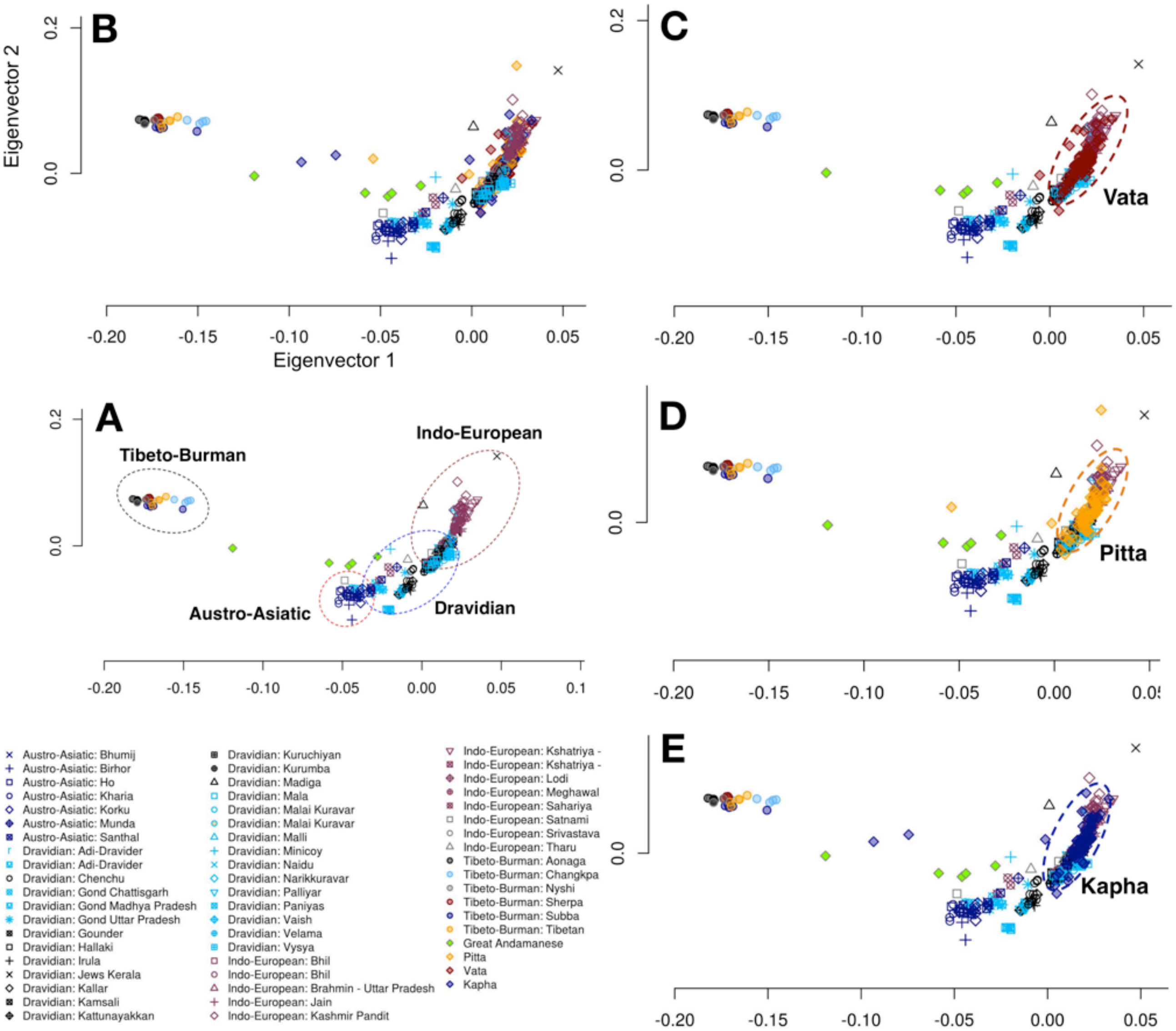

Figure S4. Tracing the ancerstry of *Prakriti* individuals. Analysis reveals that majority of the *Prakriti* samples clusters with the Dravidian, and Indo-European linguistic groups. A). PCA analysis of Indian samples with all the three *Prakriti* samples. B, C, D and E are PCA plots on eigen1 and eigen2 generated in A**.** B)**.** PCA of 297 Indian population samples. C). PCA of Indian samples with Vata. D). PCA of Indian samples with Pitta. E). PCA of Indian samples with Kapha.

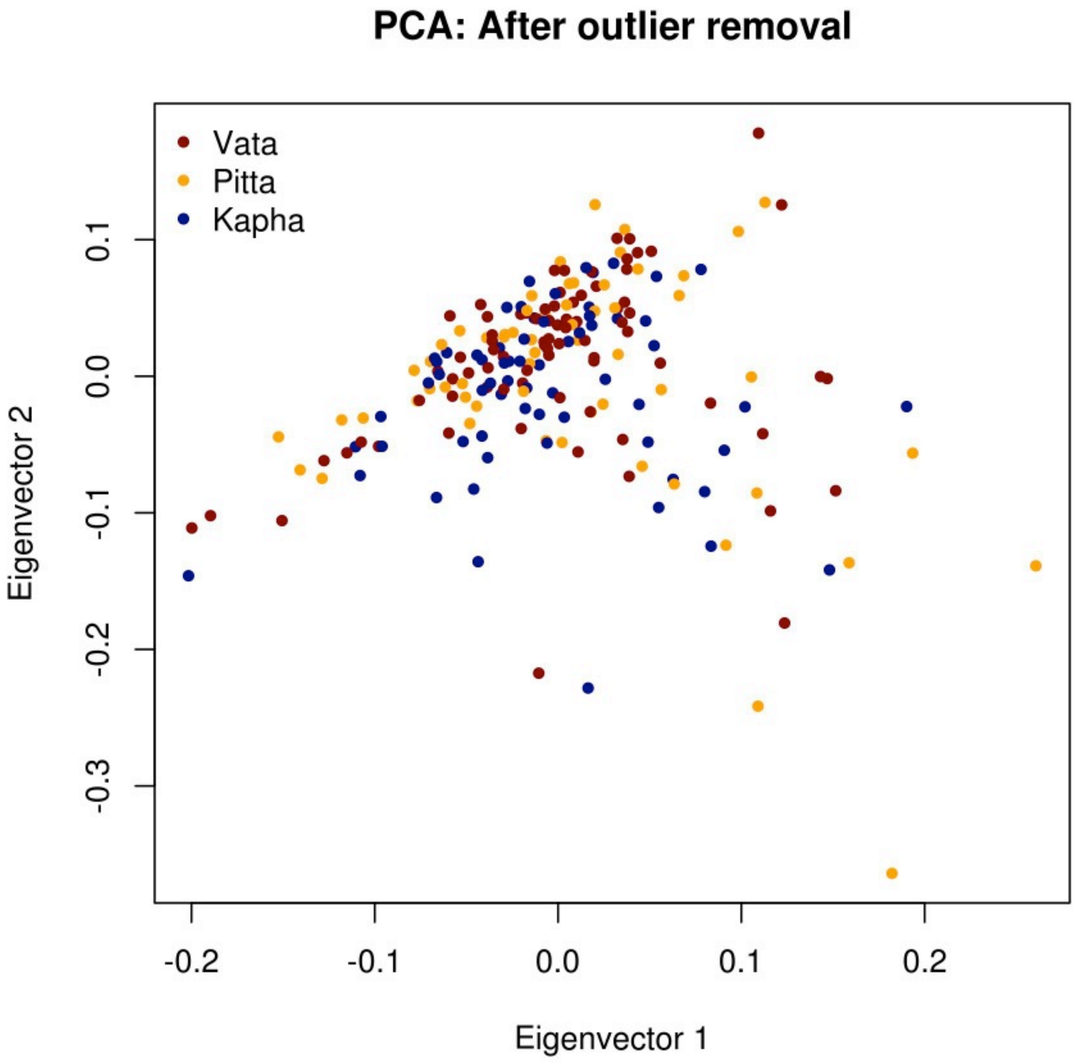

Figure S5. PCA of 205 *Prakriti* individuals after removal of 40 outlier samples.

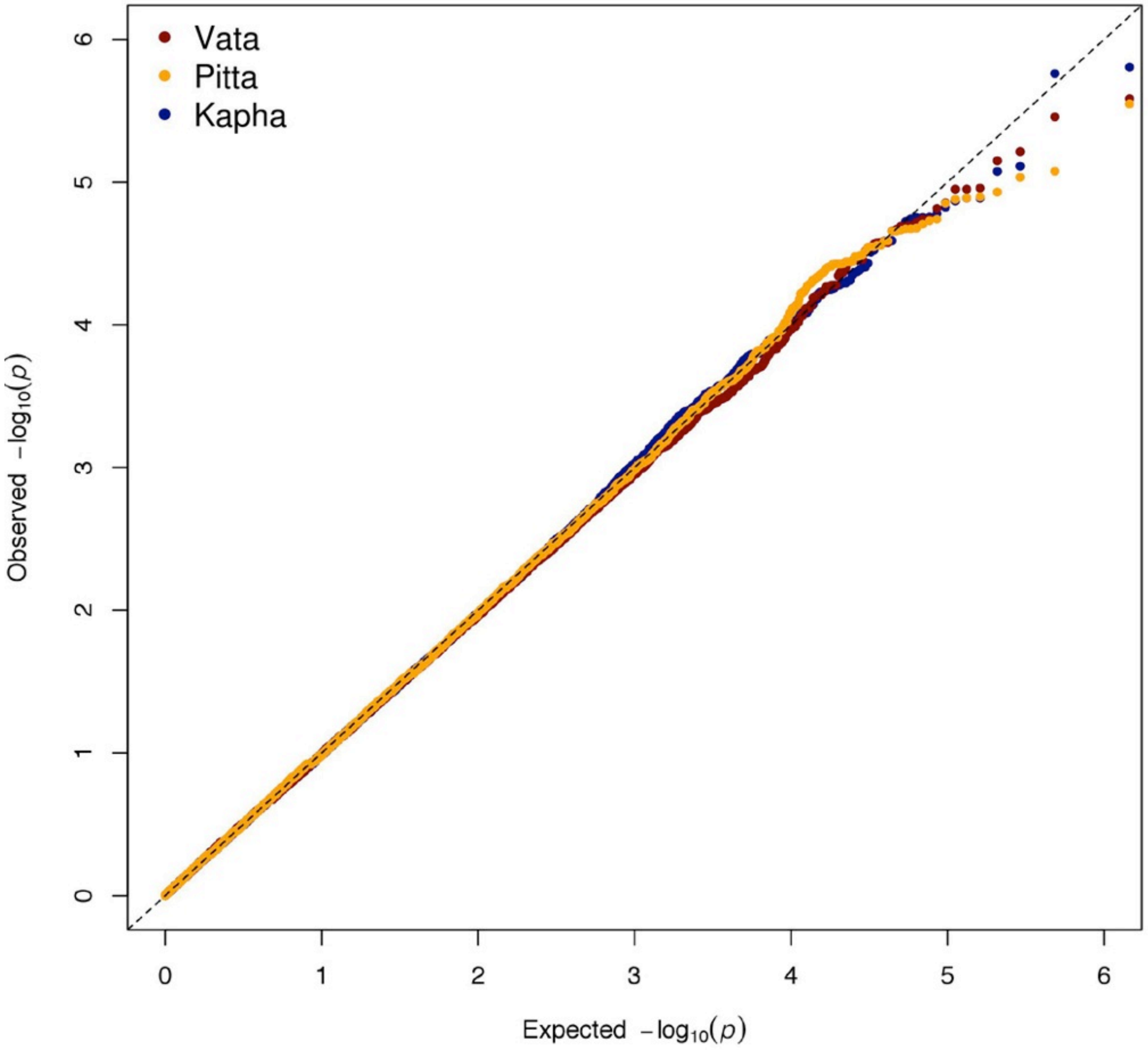

Figure S6. QQ plot of genome-wide association analysis. Deviation from expected p-valve is evident only in the tail distribution.

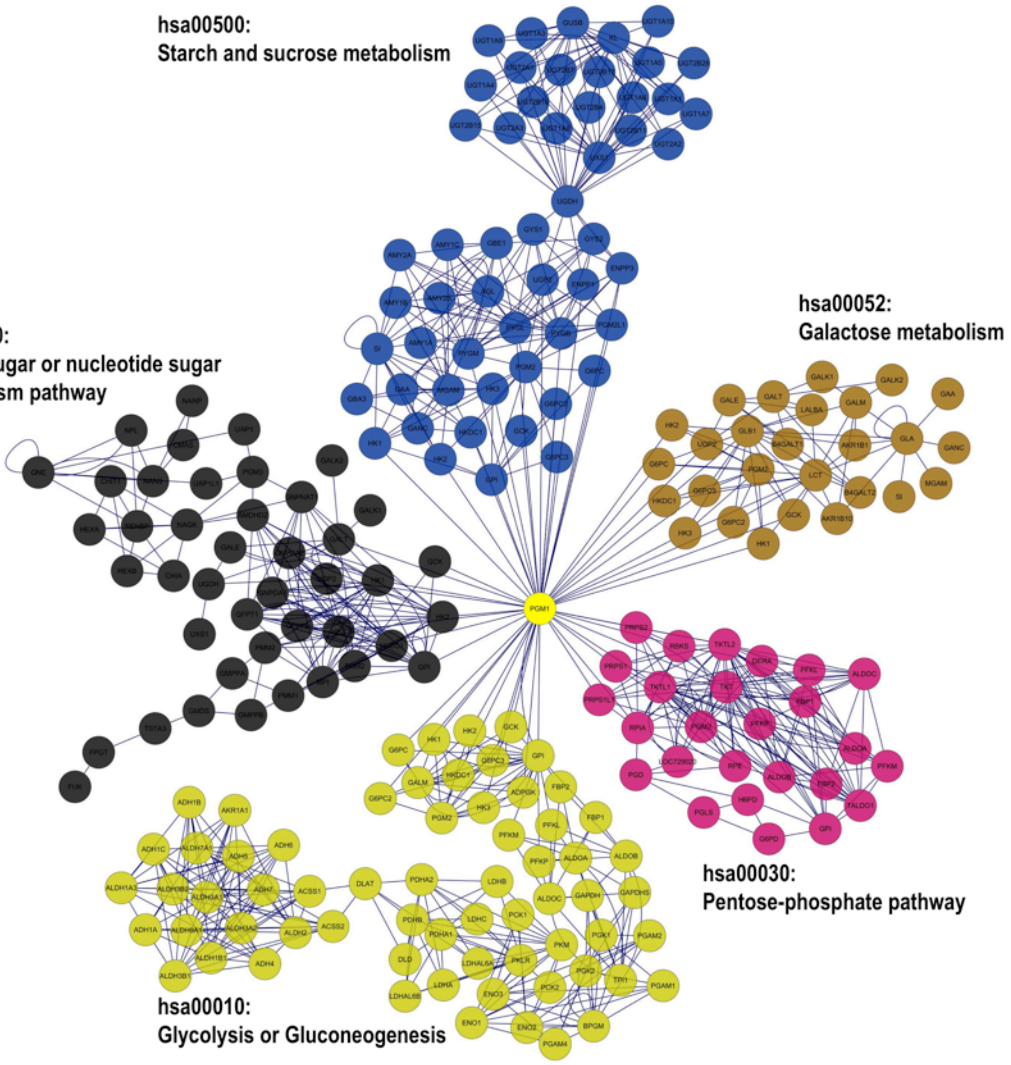

Figure S7. KEGG pathway analysis of *PGM1* gene.

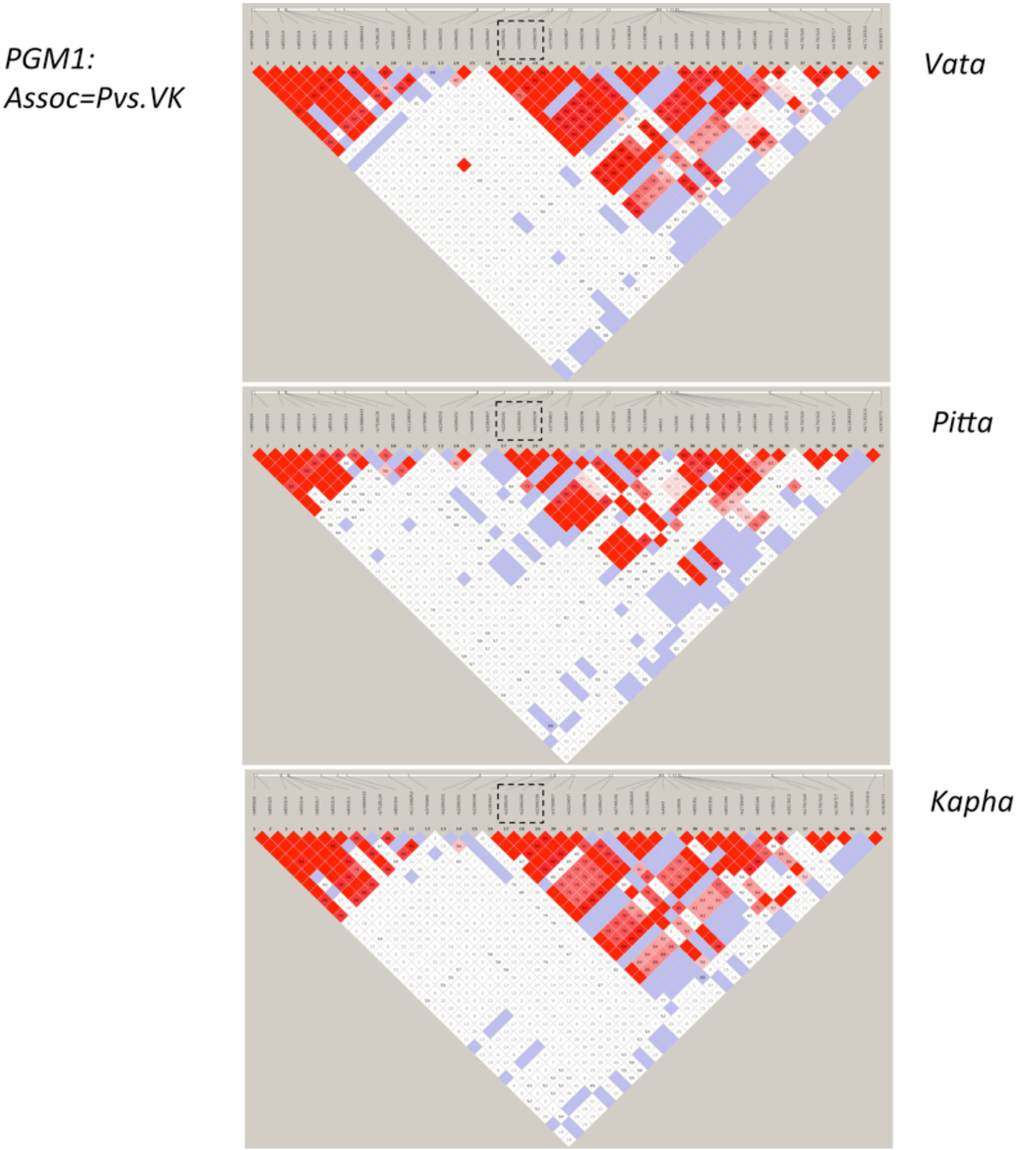

Figure S8. Linkage disequilibrium analysis for *PGM1* gene.

Supplementary Tables

Table S1. The details of the *Prakriti* individuals, who were subjected to genome-wide SNP analysis using Affymetrix (6.0) array.P - Pitta; K –Kapha; V – Vata.

S. No	Sample ID	Prakriti	First Dominant (%)	Second Dominant (%)	Third Dominant (%)	Gender	BMI	Linguistic group
1	B004	Kapha	62	P26	V12	Male	22	-
2	B014	Kapha	82	P14	V4	Male	29	-
3	B016	Kapha	87	V7	P6	Male	20.8	-
4	B018	Kapha	67	P18	V15	Male	19.4	Dravidian
5	B020	Kapha	64	P19	V17	Male	17.6	-
6	B042	Kapha	78	P18	V4	Male	24.1	Indo-European
7	B051	Kapha	79	P17	K4	Male	26.1	Indo-European
8	B053	Kapha	80	P15	V5	Male	27.8	Dravidian
9	B056	Kapha	78	V12	P10	Male	20.7	Dravidian
10	B059	Kapha	76	P16	V8	Male	21.9	Dravidian
11	B128	Kapha	74	V16	P10	Male	22.5	Dravidian
12	B157	Kapha	83	V10	P7	Male	23.5	Dravidian
13	B161	Kapha	62	P28	V10	Male	23	Dravidian
14	B185	Kapha	81	P15	V4	Male	22	Indo-European
15	B186	Kapha	85	V8	P7	Male	22.5	Dravidian
16	B205	Kapha	77	V12	P11	Male	21.3	Dravidian
17	B206	Kapha	78	P16	V6	Male	24.9	Dravidian
18	B209	Kapha	75	V17	P8	Male	25.6	Dravidian
19	B211	Kapha	70	V27	P3	Male	26.4	Dravidian
20	B213	Kapha	88	V10	P2	Male	22.9	Dravidian
21	B215	Kapha	64	P34	V2	Male	24.8	Dravidian
22	B257	Kapha	70	P18	V12	Male	25.6	Dravidian
23	B260	Kapha	72	P15	V13	Male	26.5	Dravidian
24	B262	Kapha	64	V19	P17	Male	22.2	Indo-European
25	B263	Kapha	75	P17	V8	Male	28	Indo-European
26	B269	Kapha	65	V21	P14	Male	29.6	Dravidian
27	B272	Kapha	68	V17	P15	Male	28.4	Dravidian
28	B275	Kapha	73	V20	P7	Male	22.6	Indo-European
29	B276	Kapha	60	V24	K16	Male	25.4	Dravidian
30	B277	Kapha	65	P21	V14	Male	23.3	Dravidian
31	B279	Kapha	60	V22	P18	Male	21.1	Dravidian
32	B280	Kapha	73	P24	V3	Male	24.1	Dravidian
33	B281	Kapha	74	P19	V7	Male	24.6	Dravidian
34	B283	Kapha	63	P19	V18	Male	22	Tibeto-Burman
35	B284	Kapha	60	V26	P14	Male	18.6	Tibeto-Burman
36	B285	Kapha	60	P33	V 13	Male	19.9	Dravidian
37	B290	Kapha	60	V26	P14	Male	19.3	Dravidian
38	P1043	Kapha	67	V27	P6	Male	21.3	Indo-European
39	P668	Kapha	67	P21	V12	Male	23.9	Indo-European
40	P848	Kapha	68	P20	V12	Male	25.6	Indo-European
41	P442	Kapha	66	P21	V13	Male	21.4	Indo-European
42	P542	Kapha	69	P21	V10	Male	23.7	Indo-European
43	P607	Kapha	73	V14	P13	Male	23.8	Indo-European
44	P618	Kapha	69	P24	V7	Male	25.6	Indo-European
45	P654	Kapha	71	P17	V12	Male	23.1	Indo-European
46	P668	Kapha	67	P21	V12	Male	23.9	Indo-European
47	P721	Kapha	70	V15	P15	Male	27.7	Indo-European
48	P721	Kapha	70	V15	P15	Male	27.7	Indo-European
49	P757	Kapha	74	P18	V8	Male	26.1	Indo-European
50	P773	Kapha	73	P16	V11	Male	28.7	Indo-European
51	P774	Kapha	75	P16	V9	Male	19.6	Indo-European
52	P817	Kapha	69	P18	V13	Male	33.4	Indo-European
53	P1045	Kapha	64	V19	P17	Male	18.9	Indo-European
54	P1053	Kapha	66	V25	P9	Male	26.1	Indo-European
55	P1055	Kapha	63	V25	P12	Male	26.2	Indo-European
56	P1087	Kapha	62	P24	V14	Male	25.2	Indo-European
57	P1096	Kapha	70	P22	V8	Male	-	-
58	P1099	Kapha	61	P21	V18	Male	23.3	Indo-European
59	P1100	Kapha	63	P22	V15	Male	25.1	Indo-European
60	P1111	Kapha	67	P23	V10	Male	23.5	Indo-European
61	P1115	Kapha	63	V23	P14	Male	19.8	Indo-European

62	P1142	Kapha	63	V23	P14	Male	24.3	Indo-European
63	P1159	Kapha	63	P19	V18	Male	21.6	Indo-European
64	P1164	Kapha	61	P21	V13	Male	23.5	Indo-European
65	P1183	Kapha	63	P31	V6	Male	23.4	Indo-European
66	P1210	Kapha	62	P20	V18	Male	20.7	Indo-European
67	P1252	Kapha	66	V21	P13	Male	26.3	Indo-European
68	P1256	Kapha	64	V23	P13	Male	26.8	Indo-European
69	P018	Kapha	75	P18	V7	Male	24.1	Indo-European
70	U012	Kapha	71	V15	P14	Male	24.48	Dravidian
71	U015	Kapha	72	P22	V6	Male	24.39	Dravidian
72	U020	Kapha	76	V14	P10	Male	25.2	Dravidian
73	U040	Kapha	79	P15	V6	Male	23.93	Dravidian
74	U051	Kapha	63	P28	V9	Male	20.6	Indo-European
75	U070	Kapha	75	P13	V12	Male	23.72	Dravidian
76	U071	Kapha	61	P21	V18	Male	23.93	Dravidian
77	U078	Kapha	60	P21	V19	Male	23.03	Dravidian
78	U083	Kapha	60	P24	V16	Male	23.19	Dravidian
79	U104	Kapha	70	P21	V9	Male	25.39	Dravidian
80	U113	Kapha	73	P18	V9	Male	23.93	Dravidian
81	U149	Kapha	75	P14	V11	Male	26.29	Dravidian
82	U150	Kapha	75	P13	V12	Male	22.53	Dravidian
83	U161	Kapha	73	P20	V7	Male	21.67	Indo-European
84	U168	Kapha	62	P24	V14	Male	23.38	Dravidian
85	U187	Kapha	70	P21	V9	Male	22.09	Dravidian
86	U191	Kapha	74	P15	V11	Male	26.12	Dravidian
87	U206	Kapha	61	P24	V15	Male	21.63	Indo-European
88	U210	Kapha	64	P26	V10	Male	22.22	Dravidian
89	U271	Kapha	72	P19	V9	Male	24.81	Dravidian
90	B019	Pitta	72	V17	K11	Male	19.4	-
91	B024	Pitta	67	K21	V 12	Male	23.1	-
92	B034	Pitta	67	V24	K9	Male	21.7	Indo-European
93	B041	Pitta	78	K15	V7	Male	22.1	Indo-European
94	B046	Pitta	60	K36	V4	Male	25.6	Indo-European
95	B048	Pitta	76	K15	V9	Male	26.6	Dravidian
96	B055	Pitta	84	K10	V6	Male	23.5	Dravidian
97	B060	Pitta	82	K10	V8	Male	20.2	Dravidian
98	B061	Pitta	80	V12	K8	Male	17.17	Dravidian
99	B065	Pitta	78	V26	K6	Male	22.9	Indo-European
100	B070	Pitta	82	K10	V8	Male	18	Dravidian
101	B073	Pitta	90	K6	V4	Male	21.6	Dravidian
102	B074	Pitta	78	V12	K10	Male	24.2	Indo-European
103	B076	Pitta	68	K20	V12	Male	22.7	Indo-European
104	B083	Pitta	82	K12	V6	Male	20.2	Indo-European
105	B085	Pitta	80	K12	V8	Male	26.7	Dravidian
106	B097	Pitta	80	K12	V8	Male	21.7	Indo-European
107	B100	Pitta	82	V10	K8	Male	31.7	Dravidian
108	B102	Pitta	62	K20	V18	Male	25.1	Dravidian
109	B115	Pitta	78	V12	K10	Male	22.8	Dravidian
110	B121	Pitta	86	V8	K6	Male	15	Dravidian
111	B129	Pitta	83	K12	V5	Male	29.4	Dravidian
112	B135	Pitta	82	V10	K8	Male	20.1	Dravidian
113	B140	Pitta	76	V14	K10	Male	21.2	Indo-European
114	B147	Pitta	76	K14	V10	Male	18.9	Dravidian
115	B148	Pitta	84	V10	K6	Male	20.4	Dravidian
116	B149	Pitta	82	V12	K6	Male	20.8	Dravidian
117	B154	Pitta	76	K14	V10	Male	29.6	Indo-European
118	B156	Pitta	77	K13	V10	Male	20.3	Dravidian
119	B162	Pitta	84	V10	K6	Male	17.3	Indo-European
120	B166	Pitta	76	K14	V10	Male	21.5	Dravidian
121	B169	Pitta	83	V12	K5	Male	20	Dravidian
122	B187	Pitta	74	K17	V9	Male	27.9	Indo-European
123	B191	Pitta	69	K17	V14	Male	20.5	Dravidian
124	B254	Pitta	61	K20	V19	Male	26.5	Dravidian
125	B282	Pitta	60	V23	K17	Male	20.2	Indo-European
126	P1069	Pitta	62	V18	K20	Male	20.2	-
127	P700	Pitta	65	K20	V15	Male	23.7	Indo-European
128	P788	Pitta	62	K20	V18	Male	19.8	Indo-European

129	P061	Pitta	62	V21	K17	Male	18.1	Indo-European
130	P535	Pitta	64	K31	V5	Male	23	Indo-European
131	P700	Pitta	65	K20	V15	Male	23.7	Indo-European
132	P706	Pitta	66	K22	V12	Male	21.5	Indo-European
133	P766	Pitta	63	K33	V4	Male	22.6	Indo-European
134	P788	Pitta	62	K20	V18	Male	19.8	Indo-European
135	P829	Pitta	63	K29	V8	Male	21	Indo-European
136	P835	Pitta	63	K28	V9	Male	24.1	Indo-European
137	P905	Pitta	64	K20	V16	Male	24.6	Indo-European
138	P862	Pitta	64	V19	K17	Male	16.6	Indo-European
139	P882	Pitta	63	V20	P17	Male	18.4	Indo-European
140	P1165	Pitta	62	K20	V18	Male	18.7	Indo-European
141	P1191	Pitta	60	K24	V16	Male	17	Indo-European
142	P1234	Pitta	62	K30	V8	Male	21.3	Indo-European
143	P103	Pitta	62	V21	K17	Male	19.6	Indo-European
144	P801	Pitta	63	V24	K13	Male	19.5	Indo-European
145	U034	Pitta	71	K22	V7	Male	23.8	Indo-European
146	U048	Pitta	64	K29	V7	Male	19.37	Dravidian
147	U052	Pitta	71	V15	K14	Male	21.7	Dravidian
148	U181	Pitta	63	K29	V8	Male	21.35	Dravidian
149	U190	Pitta	61	K28	V11	Male	20.82	Dravidian
150	U193	Pitta	65	K24	V11	Male	22.3	Dravidian
151	U194	Pitta	62	V22	K16	Male	18.16	Dravidian
152	U195	Pitta	61	K26	V13	Male	23.78	Dravidian
153	U221	Pitta	63	V19	K18	Male	19.46	Dravidian
154	U222	Pitta	65	K24	V11	Male	20.02	Dravidian
155	U223	Pitta	62	K26	V12	Male	19.71	Dravidian
156	U226	Pitta	67	K17	V16	Male	19.46	Dravidian
157	U238	Pitta	64	K23	V13	Male	16.79	Dravidian
158	U272	Pitta	63	V21	K16	Male	20.31	Dravidian
159	U288	Pitta	63	V19	K18	Male	20.76	Dravidian
160	U303	Pitta	63	V25	K12	Male	20.76	Dravidian
161	U315	Pitta	63	K19	V18	Male	20.19	Dravidian
162	B114	Vata	86	P12	K2	Male	22.9	Dravidian
163	B033	Vata	75	P15	K10	Male	15.6	Dravidian
164	B038	Vata	80	K14	P6	Male	17.7	Indo-European
165	B045	Vata	82	P12	K6	Male	20.1	Dravidian
166	B047	Vata	79	P13	K8	Male	-	Dravidian
167	B052	Vata	73	K15	P12	Male	20.5	Dravidian
168	B062	Vata	84	P9	K7	Male	21.3	Dravidian
169	B063	Vata	82	K10	V8	Male	24.2	Dravidian
170	B071	Vata	78	P12	K10	Male	17.9	Dravidian
171	B077	Vata	76	P14	K10	Male	21	Dravidian
172	B078	Vata	84	K10	P6	Male	22.7	Dravidian
173	B081	Vata	78	K14	P8	Male	20.6	Dravidian
174	B084	Vata	76	P14	K10	Male	21.1	Indo-European
175	B089	Vata	82	P12	K6	Male	20.2	Dravidian
176	B099	Vata	76	K16	P8	Male	20.5	Dravidian
177	B103	Vata	84	P10	K6	Male	21.5	Indo-European
178	B107	Vata	82	P12	K6	Male	15.4	Indo-European
179	B119	Vata	76	P12	K12	Male	18.5	Dravidian
180	B120	Vata	82	K10	P8	Male	18.1	Dravidian
181	B123	Vata	76	P14	K10	Male	20.3	Indo-European
182	B126	Vata	78	P12	K10	Male	19	Dravidian
183	B143	Vata	72	P18	K10	Male	23.3	Dravidian
184	B160	Vata	76	P18	K6	Male	16.1	Dravidian
185	B168	Vata	82	K10	P8	Male	19.8	Dravidian
186	B190	Vata	78	K13	P9	Male	17.6	Dravidian
187	B193	Vata	79	K18	P3	Male	20	Dravidian
188	B199	Vata	75	P17	K8	Male	23.4	Dravidian
189	B210	Vata	73	P14	K13	Male	21.5	Dravidian
190	B212	Vata	67	K26	P7	Male	21.5	-
191	B244	Vata	60	P25	K15	Male	20.1	Indo-European
192	B247	Vata	60	K29	P11	Male	18.1	Indo-European
193	B255	Vata	67	P19	K14	Male	20.2	Dravidian
194	B259	Vata	64	K23	P13	Male	18.1	Dravidian
195	B261	Vata	60	K24	P16	Male	20.2	Dravidian

196	B264	Vata	60	P23	K17	Male	19.3	Indo-European
197	B266	Vata	60	K33	P7	Male	25.8	Indo-European
198	B271	Vata	60	K26	P14	Male	18.1	Dravidian
199	B289	Vata	60	K21	P19	Male	17.3	-
200	P1004	Vata	68	K26	P6	Male	16.9	Indo-European
201	P1031	Vata	65	P24	K11	Male	19.1	Dravidian
202	P1040	Vata	66	K30	P4	Male	17.9	Indo-European
203	P1073	Vata	65	K19	P16	Male	20.2	Indo-European
204	P335	Vata	72	P17	K11	Male	16.7	Indo-European
205	P421	Vata	73	K15	V13	Male	20.9	Indo-European
206	P452	Vata	71	P18	K11	Male	18.3	Indo-European
207	P487	Vata	73	K17	P10	Male	19.1	Indo-European
208	P606	Vata	69	K22	P9	Male	19.1	Indo-European
209	P653	Vata	72	K21	V7	Male	16.4	Indo-European
210	P707	Vata	69	P21	K10	Male	22.2	Indo-European
211	P728	Vata	71	K17	P12	Male	18.5	Indo-European
212	P776	Vata	74	K18	V8	Male	20	Dravidian
213	P810	Vata	68	K27	P5	Male	24.1	Indo-European
214	P1023	Vata	64	K28	P8	Male	16.5	Indo-European
215	P1033	Vata	64	K23	P13	Male	15.4	Indo-European
216	P1036	Vata	62	K24	P14	Male	22	Indo-European
217	P1040	Vata	66	K30	P4	Male	17.9	Indo-European
218	P993	Vata	66	K24	P10	Male	18	Indo-European
219	P1047	Vata	63	K28	P9	Male	27.1	Indo-European
220	P1048	Vata	64	K21	P15	Male	23.1	Indo-European
221	P1049	Vata	62	P21	P17	Male	17.7	Indo-European
222	P1062	Vata	62	K26	P12	Male	21.1	Indo-European
223	P1083	Vata	69	K20	P11	Male	22.3	Indo-European
224	P1093	Vata	66	P21	K13	Male	21.4	Indo-European
225	P1094	Vata	63	K22	P15	Male	-	-
226	P1101	Vata	65	K24	P11	Male	20.7	Indo-European
227	P1105	Vata	67	K34	P9	Male	19.7	Indo-European
228	P1107	Vata	62	K25	P13	Male	24	Indo-European
229	P1108	Vata	66	K26	P8	Male	19	Indo-European
230	P1114	Vata	63	K28	P9	Male	19.8	Indo-European
231	P1126	Vata	63	K32	P5	Male	27.3	Indo-European
232	P1128	Vata	60	K23	P17	Male	17.9	Indo-European
233	P1133	Vata	67	K24	P9	Male	22.8	Indo-European
234	P1135	Vata	62	K27	P11	Male	19	Indo-European
235	P1149	Vata	66	K27	P7	Male	19.1	Indo-European
236	P1181	Vata	60	K27	P13	Male	19.5	Indo-European
237	P1258	Vata	61	K20	P19	Male	17.6	Indo-European
238	P1259	Vata	61	k20	P19	Male	21.3	Indo-European
239	P055	Vata	79	P16	K5	Male	15.8	Indo-European
240	P183	Vata	73	P15	K12	Male	18.3	Indo-European
241	P214	Vata	78	K 14	P8	Male	17.7	Indo-European
242	U014	Vata	63	P22	K15	Male	20.2	Dravidian
243	U049	Vata	64	K21	P15	Male	14.01	Dravidian
244	U076	Vata	65	K20	P15	Male	16.33	Dravidian
245	U114	Vata	63	K23	P14	Male	17.99	Dravidian
246	U120	Vata	63	K25	P12	Male	20.81	Dravidian
247	U124	Vata	64	K22	P14	Male	17.48	Dravidian
248	U146	Vata	66	K19	P15	Male	19.85	Dravidian
249	U165	Vata	62	K22	P16	Male	22.38	Dravidian
250	U202	Vata	65	P21	K14	Male	19.83	Dravidian
251	U247	Vata	67	K20	P13	Male	17.95	Dravidian
252	U309	Vata	60	P25	K15	Male	20.06	Dravidian
253	B101	Pitta	80	V10	K10	Male	31.7	Dravidian
254	B102	Pitta	62	K20	V18	Male	25.1	Dravidian
255	B131	Pitta	68	K20	V12	Male	25.0	Dravidian
256	B202	Kapha	93	V6	P1	Male	23.9	Dravidian
257	B287	Vata	70	K20	P10	Male	22.0	Dravidian
258	B288	Kapha	68	P18	V14	Male	22.9	Dravidian
259	U009	Kapha	71	P23	V6	Male	28.72	Indo-European
260	U056	Pitta	64	K31	V5	Male	22.73	Indo-European
261	U069	Kapha	70	P19	V11	Male	24.09	Indo-European
262	U101	Vata	64	K30	P6	Male	19.88	Dravidian

Table S2. The details of genotype call rates of individual *Prakriti* samples.

Sample ID	Genotype frequency	Sample ID	Genotype frequency	Sample ID	Genotype frequency	Sample ID	Genotype frequency	Sample ID	Genotype frequency	Sample ID	Genotype frequency	Sample ID	Genotype frequency
U315	0.9790	U049	0.9863	P1107	0.9874	P700	0.9677	B261	0.9770	B126	0.9434	B041	0.9624
U309	0.9526	U048	0.9598	P1105	0.9886	P668	0.9452	B260	0.9466	B123	0.9702	B038	0.9755
U303	0.9812	U040	0.9618	P1101	0.9835	P654	0.9894	B259	0.9467	B121	0.9598	B034	0.9655
U288	0.9629	U034	0.9769	P1100	0.9688	P653	0.9904	B257	0.9471	B120	0.9439	B033	0.9652
U272	0.9838	U020	0.9711	P1099	0.9851	P618	0.9886	B255	0.9442	B119	0.9611	B024	0.9580
U271	0.9683	U015	0.9799	P1096	0.9790	P607	0.9877	B254	0.9578	B115	0.9535	B020	0.9707
U247	0.9707	U014	0.9672	P1094	0.9680	P606	0.9722	B247	0.9617	B107	0.9758	B019	0.9375
U238	0.9640	U012	0.9533	P1093	0.9896	P542	0.9683	B244	0.9531	B103	0.9486	B018	0.9798
U226	0.9547	P1073	0.9855	P1087	0.9729	P535	0.9843	B215	0.9524	B102	0.9234	B016	0.9624
U223	0.9413	P1069	0.9823	P1083	0.9776	P487	0.9755	B213	0.9528	B100	0.9584	B014	0.9581
U222	0.9682	P1043	0.9794	P1062	0.9808	P452	0.9508	B212	0.9601	B099	0.9579	B114	0.9503
U221	0.8928	P1040	0.9865	P1055	0.9718	P442	0.9800	B211	0.9573	B097	0.9699	B004	0.9667
U210	0.9454	P1031	0.9869	P1053	0.9886	P421	0.9905	B210	0.9607	B089	0.9521	B046	0.9488
U206	0.9786	P1004	0.9783	P1049	0.9760	P335	0.9898	B209	0.9294	B085	0.9625	B045	0.9657
U202	0.9667	P848	0.9841	P1048	0.9768	P214	0.9591	B206	0.9456	B084	0.9453	B042	0.9599
U195	0.9603	P788	0.9757	P1047	0.9901	P183	0.9886	B205	0.9632	B083	0.9761	B135	0.9666
U194	0.9759	P700	0.9845	P1045	0.9732	P801	0.9834	B199	0.9518	B081	0.9632	B129	0.9635
U193	0.9796	P668	0.9868	P1040	0.9730	P103	0.9585	B193	0.9878	B078	0.9510	B128	0.9433
U191	0.9741	P1259	0.9706	P1036	0.9883	P055	0.9762	B191	0.9531	B077	0.9629	B264	0.9725
U190	0.9824	P1258	0.9762	P1033	0.9716	P018	0.9661	B190	0.9537	B076	0.9225	B263	0.9755
U187	0.9683	P1256	0.9810	P1023	0.9553	P061	0.9859	B187	0.9752	B074	0.9483	B262	0.9641
U181	0.9586	P1252	0.9872	P993	0.9679	B290	0.9477	B186	0.9804	B073	0.9677	P721	0.9819
U168	0.9816	P1234	0.9741	P882	0.9640	B289	0.9828	B185	0.9751	B071	0.9807	P707	0.9647
U165	0.9824	P1210	0.9659	P862	0.9484	B285	0.9795	B169	0.9483	B070	0.9799	P706	0.9610
U161	0.9764	P1191	0.9225	P905	0.9739	B284	0.9585	B168	0.9568	B065	0.9482	P1114	0.9924
U150	0.9674	P1183	0.9831	P835	0.9753	B283	0.9669	B166	0.9512	B063	0.9337	P1111	0.9719
U149	0.9424	P1181	0.9648	P829	0.9734	B282	0.9801	B162	0.9644	B062	0.9586	P1108	0.9795
U146	0.9649	P1165	0.9280	P817	0.9430	B281	0.9787	B161	0.9865	B061	0.9583	U070	0.9891
U124	0.9485	P1164	0.9340	P810	0.9628	B280	0.9543	B160	0.9554	B060	0.9610	U052	0.9822
U120	0.9670	P1159	0.9526	P788	0.9451	B279	0.9474	B157	0.9745	B059	0.9676	U051	0.9808
U114	0.9722	P1149	0.9822	P776	0.9541	B277	0.9475	B156	0.9610	B056	0.9680	U009	0.8913
U113	0.9461	P1142	0.9574	P774	0.9687	B276	0.9447	B154	0.9702	B055	0.9275	U056	0.9147
U104	0.9854	P1135	0.9783	P773	0.9518	B275	0.9407	B149	0.9537	B053	0.9716	U069	0.9113
U083	0.9642	P1133	0.9371	P766	0.9866	B272	0.9632	B148	0.9685	B052	0.9415	U101	0.8977
U078	0.9659	P1128	0.9670	P757	0.9916	B271	0.9445	B147	0.9507	B051	0.9457	B101	0.8977
U076	0.9668	P1126	0.9791	P728	0.9880	B269	0.9699	B143	0.9784	B048	0.9670	B102	0.9412
U071	0.9847	P1115	0.9845	P721	0.9602	B266	0.9278	B140	0.9434	B047	0.9524	B131	0.9153
B202	0.9109	B287	0.9207	B288	0.9528	-		-		-		-	

Table S3. The list of randomly selected 48 markers from Affymetrix SNP (6.0) array for validation of 48 known genotyped individuals using custom-designed VeraCode GoldenGate Genotyping.

S. No	rsID	Chr	Position	% of genotype not matching (no of individuals)	% of genotype matching (no of individuals)	% of genotype not called
1	rs12060189	1	40932868	0 (0)	1 (48)	0 (0)
2	rs10489167	1	40949053	0 (0)	1 (48)	0 (0)
3	rs2744803	1	40955692	0 (0)	1 (48)	0 (0)
4	rs823681	1	41004011	0 (0)	1 (48)	0 (0)
5	rs6713149	2	2441676	0 (0)	1 (48)	0 (0)
6	rs4853937	2	2443758	0 (0)	1 (48)	0 (0)
7	rs11127348	2	2473921	0 (0)	1 (48)	0 (0)
8	rs6748662	2	57710603	0 (0)	1 (48)	0 (0)
9	rs6733573	2	204840871	0 (0)	1 (48)	0 (0)
10	rs12490846	3	61700707	0 (0)	1 (48)	0 (0)
11	rs10511210	3	104538562	0.0208 (1)	0.9791 (47)	0 (0)
12	rs16870624	4	20864143	0 (0)	1 (48)	0 (0)
13	rs17627904	4	25584319	0 (0)	1 (48)	0 (0)
14	rs17797407	4	56485560	0 (0)	1 (48)	0 (0)
15	rs7665845	4	162413989	0.0208(1)	0.9791(47)	0 (0)
16	rs4699948	5	59312197	0 (0)	1 (48)	0 (0)
17	rs152270	5	141123895	0 (0)	1 (48)	0 (0)
18	rs248478	5	141139422	0 (0)	1 (48)	0 (0)
19	rs429946	5	141164104	0.0208 (1)	0.9791 (47)	0 (0)
20	rs790607	6	91163700	0 (0)	1 (48)	0 (0)
21	rs2717983	7	37300345	0 (0)	1 (48)	0 (0)
22	rs41028	7	103930457	0 (0)	1 (48)	0 (0)
23	rs41038	7	103933117	0 (0)	1 (48)	0 (0)
24	rs10231685	7	103963525	0 (0)	1 (48)	0 (0)
25	rs13231181	7	103979084	0 (0)	1 (48)	0 (0)
26	rs6969323	7	104001723	0 (0)	1 (48)	0 (0)
27	rs17077609	8	6430547	0 (0)	1 (48)	0 (0)
28	rs6996364	8	69154894	0 (0)	0.9791 (47)	0.0208 (1)
29	rs9299502	10	70546940	0.0208 (1)	0.9791 (47)	0 (0)
30	rs2803545	10	88396950	0 (0)	1 (48)	0 (0)
31	rs1147644	10	95390937	0 (0)	1 (48)	0 (0)
32	rs913046	10	95398474	0 (0)	1 (48)	0 (0)
33	rs6583901	10	95399988	0 (0)	1 (48)	0 (0)
34	rs12772131	10	95461814	0 (0)	1 (48)	0 (0)
35	rs3809101	11	1923847	0 (0)	1 (48)	0 (0)
36	rs11600982	11	1932882	0.0208 (1)	0.9791 (47)	0 (0)
37	rs10769945	11	1941703	0.0208 (1)	0.9791 (47)	0 (0)
38	rs3751176	12	127241860	0.0208 (1)	0.9791 (47)	0 (0)
39	rs11157525	14	46288404	0 (0)	1 (48)	0 (0)
40	rs11848659	14	46293695	0 (0)	1 (48)	0 (0)
41	rs2105269	14	68816987	0 (0)	1 (48)	0 (0)
42	rs4902778	14	69649989	0 (0)	1 (48)	0 (0)
43	rs7162785	15	91094879	0 (0)	1 (48)	0 (0)
44	rs11247168	15	98578034	0 (0)	1 (48)	0 (0)
45	rs4789092	17	70203226	0.0208 (1)	0.9791 (47)	0 (0)
46	rs1077053	20	30663242	0 (0)	1 (48)	0 (0)
47	rs2835193	21	36268715	0 (0)	1 (48)	0 (0)
48	rs2835216	21	36285255	0 (0)	1 (48)	0 (0)

Table S4. Genotype imputation of 2%, 5% and 10% masked data with different reference populations (India, HapMap and India+HapMap) and their concordance frequency.

Simulation	2% mask data (concordance frequency)				5% mask data (concordance frequency)				10% mask data (concordance frequency)			
	India (IN)	HapMap (HM)	India+HapMap (IH)	1000 genme proj. - South-Asians (SA)	India (IN)	HapMap (HM)	India+HapMap (IH)	1000 genme proj. - South-Asians (SA)	India (IN)	HapMap (HM)	India+HapMap (IH)	1000 genme proj. - South-Asians (SA)
1st	0.951330459	0.948937737	0.948280395	0.949794864	0.95073382	0.943192969	0.944974188	0.947707348	0.948816529	0.940164723	0.942259314	0.94384211
2nd	0.951863232	0.944536359	0.944956538	0.948337689	0.950245628	0.943649364	0.944949436	0.947475941	0.946401719	0.938915895	0.941332794	0.94487523
3rd	0.952389701	0.945723209	0.945828193	0.949200462	0.94990249	0.948163587	0.945363422	0.946516295	0.94642584	0.938645949	0.940803995	0.94298547
4th	0.952344714	0.945994174	0.947647414	0.947084332	0.950430667	0.942883336	0.944066604	0.948107471	0.947232722	0.940104224	0.941819433	0.94467963
5th	0.951133647	0.94516985	0.945747839	0.948545929	0.949526074	0.942946621	0.945249175	0.944344858	0.947550272	0.940470732	NA	0.944215615
6th	0.950966253	0.945590896	0.946534861	0.949032219	0.951297975	0.94493859	0.945343375	0.947600831	0.947660929	0.939608344	0.941737502	0.943756911
7th	0.951654078	0.945943101	0.946706319	0.950355688	0.951453516	0.944396474	0.945963521	0.9459353	0.947956956	0.940232287	0.941211548	0.941635472
8th	0.95214682	0.945520995	0.945547288	0.948900682	0.950964827	0.943016057	0.94544544	0.947449369	0.947523537	0.938894801	0.94134534	0.943096066
9th	0.951490479	0.944924491	0.946237689	0.949304145	0.949855969	0.94318788	0.943614638	0.94691844	0.947550709	0.939662284	0.941645316	0.943706757
10th	0.951990324	0.944681075	0.945837934	0.949192106	0.949135007	0.941807632	0.94332217	0.947997587	0.947700952	0.939114704	0.942093383	0.944512862
11th	0.952354702	0.947183283	0.947367039	0.946834982	0.950930644	0.94292476	0.944555785	0.94714827	0.94781849	0.940779774	0.942166767	0.943066331
12th	0.950539781	0.945496572	0.944708571	0.949880089	0.949561824	0.943069149	0.944188575	0.947506303	0.947704772	0.939717805	0.941357839	0.944972301
13th	0.950464884	0.942585491	0.943268372	0.9468386	0.951002655	0.943273526	0.946045351	0.945784766	0.948994375	0.939859284	0.942887913	0.94366507
14th	0.950637695	0.946622579	0.947016218	0.947656559	0.951827384	0.945490374	0.947375883	0.945770893	0.947701749	0.939785216	0.941051861	0.944326411
15th	0.953131154	0.945989235	0.947039517	0.948803595	0.951064533	0.943505656	0.945296758	0.947549907	0.947867532	0.93967435	0.942385369	0.943734273
16th	0.952325917	0.944918705	0.943526569	0.947394087	0.95061847	0.943958847	0.944684575	0.947333473	0.947995299	0.939593867	0.942337861	0.943822134
17th	0.951144597	0.946025412	0.945631629	0.947732864	0.949601585	0.943433612	0.944765212	0.948186528	0.946968745	0.938724781	0.94116683	0.943856538
18th	0.950341387	0.946769958	0.946323529	0.946786203	0.950588963	0.943173573	0.944571294	0.94541224	0.947742612	0.938885613	0.941643186	0.944537731
19th	0.952583603	0.949168571	0.948117792	0.947609792	0.952873916	0.94724692	0.94986203	0.947509763	0.9476191	0.940534842	0.942610415	0.943976112
20th	0.951543469	0.946424822	0.945794834	0.950414478	0.949269104	0.941016537	0.944023286	0.947401493	0.947431968	0.940160294	0.941848459	0.944341758
21st	0.953079872	0.947618547	0.948012393	0.945890931	0.948871394	0.941169568	0.936475934	0.9457487	0.947820907	0.939797558	0.942175049	0.944325751
22nd	0.950999711	0.946664565	0.945718715	0.949040713	0.94984062	0.942921717	0.943795908	0.946763937	0.94857146	0.939417442	0.94252522	0.945703009
23rd	0.951310468	0.945926782	0.946031829	0.947881845	0.950062382	0.943930815	0.945594335	0.947259845	0.948212118	0.940232551	0.942677284	0.943587777
24th	0.951339286	0.945063025	0.947111345	0.949379005	0.950555781	0.943429839	0.944944635	0.947804331	0.947798206	0.940260435	0.941975551	0.94467946
25th	0.950723648	0.946442174	0.945890573	0.950109316	0.951890584	0.94362894	0.945760977	0.94715687	0.94808325	0.940464349	0.941562913	0.943935229
26th	0.949868766	0.944173228	0.945800525	0.948355093	0.950783284	0.943387971	0.945340846	0.948218949	0.947968697	0.939634726	0.941924519	0.943810194

27th	0.95150528	0.946146167	0.944097095	0.948475193	0.949792933	0.942755853	0.944618922	0.947442867	0.94751505	0.938802154	0.94248943	0.943276268
28th	0.952910039	0.947223605	0.947040172	0.949867994	0.948920204	0.942236766	0.943974247	0.948439002	0.947784827	0.940430039	0.94266323	0.944046272
29th	0.952079613	0.944858733	0.945672723	0.949492802	0.95054353	0.942979976	0.944718847	0.946606903	0.947610877	0.939528733	0.941594352	0.943640882
30th	0.952305956	0.946189989	0.946294984	0.946478531	0.950348181	0.942371472	0.944408305	0.948639703	0.947684073	0.939310684	0.941429976	0.944605827
31st	0.950701266	0.944213899	0.944870515	0.947657316	0.950630442	0.941933215	0.944992166	0.947427472	0.946948601	0.938961074	0.940925131	0.944370078
32nd	0.951885702	0.946186574	0.945635046	0.949097879	0.949267253	0.944432816	0.94397291	0.947828331	0.948180527	0.939760682	0.941744072	0.944787473
33rd	0.952687438	0.94654022	0.946776651	0.949282413	0.952557975	0.944547708	0.946878504	0.948005417	0.947460442	0.93957623	0.941674316	0.944307445
34th	0.950751208	0.944841353	0.946154654	0.946957973	0.952590015	0.945341838	0.947371328	0.947737036	0.947656459	0.939078329	0.941243364	0.943476867
35th	0.951367223	0.944550769	0.945415935	0.94826644	0.949401808	0.9417283	0.942560753	0.948794625	0.946818167	0.938621625	0.941153312	0.943961966
36th	0.954613506	0.948178499	0.948677541	0.948292653	0.950555544	0.943784522	0.944648227	0.948268864	0.947411268	0.939816636	0.942116881	0.94387723
37th	0.951683594	0.94562108	0.946434664	0.946839822	0.950883355	0.944593444	0.9449553	0.947995466	0.947941254	0.940849288	0.942248011	0.943718833
38th	0.952188411	0.946622207	0.946595951	0.946085425	0.94993602	0.942482406	0.944071231	0.946095191	0.94709336	0.938860523	0.940932399	0.944089427
39th	0.95271762	0.94612069	0.947119428	0.947304517	0.949872096	0.942315071	0.944254956	0.946978567	0.946728829	0.938998559	0.940881993	0.944372883
40th	0.951304667	0.944978212	0.945923243	0.950226244	0.951124093	0.942862625	0.944632178	0.947111303	0.946936525	0.939800609	0.941209229	0.943665161
41st	0.952151852	0.946630912	0.946920104	0.949006864	0.950133419	0.942181663	0.944327036	0.947202677	0.947668351	0.940543642	0.941860402	0.944330818
42nd	0.950440616	0.944495594	0.945679337	0.945537938	0.950565845	0.943586028	0.94453443	0.946220698	0.948188822	0.940348577	0.942167951	0.943908202
43rd	0.951207323	0.944421064	0.94573623	0.948214449	0.948517791	0.941330998	0.942494981	0.945626681	0.94815762	0.940190276	0.942354246	0.944914881
44th	0.950815374	0.947774107	0.946699177	0.948403172	0.94951823	0.943340056	0.944513802	0.947607555	0.947471614	0.940218786	0.941469462	0.943497358
45th	0.953363512	0.946152227	0.9480998	0.947681657	0.94895087	0.941380928	0.943246759	0.946976229	0.947768001	0.939307542	0.941574224	0.943382593
46th	0.952178589	0.945279505	0.945961544	0.945329604	0.950703699	0.943672041	0.944931124	0.947657691	0.948715288	0.939852006	0.942380471	0.943282925
47th	0.94995405	0.94745963	0.945359065	0.948514671	0.949492256	0.941566753	0.943284123	0.945801189	0.947413581	0.939363301	0.941412761	0.944220105
48th	0.951654078	0.94607469	0.944732479	0.947937221	0.949643204	0.942144617	0.943349938	0.94738138	0.947004482	0.938958128	0.940669072	0.943848386
49th	0.951090097	0.946703441	0.946834778	0.9479268	0.950373663	0.943412117	0.944904639	0.944451264	0.947549159	0.940544413	0.941479472	0.943550413
50th	0.95163999	0.947362897	0.946549462	0.948542805	0.950017034	0.942075118	0.944002044	0.946913109	0.947655832	0.93971283	0.941960438	0.9449498
51st	0.953439542	0.947560431	0.946799297	0.947793359	0.948694994	0.942705502	0.943984397	0.94634699	0.948028134	0.939330578	0.942111169	0.94463318
52nd	0.949437466	0.943326952	0.943195825	0.948607942	0.949479623	0.942277691	0.944307909	0.946773579	0.946295669	0.938907771	0.941197364	0.943624828
53rd	0.951763594	0.947065925	0.947538316	0.948371217	0.953446281	0.946740646	0.948283536	0.948316019	0.948197878	0.939162688	0.941680961	0.942972973
54th	0.951401673	0.945142797	0.945090201	0.947704664	0.949920653	0.943817831	0.944148001	0.947650875	0.946918996	0.939303216	0.941379254	0.944280805
55th	0.950605263	0.945763158	0.946342105	0.947849675	0.951844437	0.943761714	0.945582723	0.945573035	0.947225257	0.939436312	0.941511907	0.94439133
56th	0.951323722	0.945032765	0.946526868	0.948822958	0.949868973	0.942795662	0.943520037	0.946139903	0.948096206	0.94099657	0.942503877	0.943507154
57th	0.952354716	0.945244654	0.94629411	0.947406844	0.951147605	0.943971035	0.946039956	0.945842727	0.947057216	0.939330018	0.941259085	0.944337571
58th	0.950809775	0.945743759	0.945113788	0.950205074	0.950417631	0.943601122	0.94428384	0.947041955	0.948006118	0.939222113	0.941620234	0.943894389
59th	0.952644704	0.946974669	0.94726342	0.946781011	0.949299917	0.941201543	0.943918761	0.946448585	0.94836122	0.940470596	0.941956919	0.944306744
60th	0.952359657	0.947966539	0.945704214	0.949498633	0.950953942	0.943565904	0.944935597	0.946950972	0.948104065	0.93952663	0.941863449	0.943901504
61st	0.954079756	0.948957075	0.950165502	0.949388916	0.949533747	0.941521029	0.943676248	0.946829184	0.947324959	0.939400734	0.941539017	0.944064816
62nd	0.951727215	0.94450861	0.946109828	0.948597847	0.950577274	0.943733143	0.945385542	0.948318728	0.947559896	0.93938798	0.941917123	0.943770173
63rd	0.949482478	0.943913203	0.944569957	0.947800694	0.94993921	0.947312459	0.944638782	0.944252923	0.947050759	0.938994216	0.941271896	0.944125615
64th	0.951902124	0.946309958	0.946126178	0.94432199	0.95051856	0.944023255	0.944236216	0.946435154	0.947019289	0.939408499	0.941095539	0.944338572

65th	0.952747628	0.948148537	0.947018475	0.944773895	0.950868809	0.943860735	0.944724744	0.948552223	0.947353473	0.939635915	0.94165679	0.944472707
66th	0.950857563	0.945420639	0.946681375	0.950177504	0.951332104	0.94408256	0.945703046	0.947293886	0.947943111	0.93930609	0.942008572	0.943187026
67th	0.95269631	0.947046147	0.947177547	0.94785621	0.950200923	0.942323886	0.945009966	0.946572593	0.947617514	0.940313662	0.941994967	0.944986729
68th	0.953191043	0.946995353	0.947572917	0.950221709	0.951826524	0.945781394	0.94621422	0.946485987	0.948104622	0.940222471	0.942430785	0.943514868
69th	0.951638721	0.946338135	0.946626781	0.946899967	0.950347281	0.944136697	0.944839782	0.946520309	0.948320102	0.940206479	0.942947494	0.943425894
70th	0.951721055	0.947198191	0.946724868	0.948078322	0.949374507	0.943758924	0.944813844	0.946979023	0.947088356	0.939306105	0.942046026	0.944725545
71st	0.951874606	0.946124764	0.946072254	0.949738665	0.950567952	0.943247448	0.944494171	0.947124144	0.946691498	0.938635966	0.940596601	0.943196066
72nd	0.951465225	0.946087048	0.946795393	0.947365222	0.949825992	0.943196618	0.945224929	0.945932973	0.948109237	0.939784194	0.939893518	0.944315823
73rd	0.950344356	0.946322486	0.945849324	0.94815423	0.949168256	0.941282409	0.943403063	0.947417678	0.9473141	0.939030051	0.941072394	0.94361656
74th	0.953987199	0.949532953	0.95052759	0.948835515	0.951157714	0.9444661	0.945492581	0.947449939	0.94750339	0.9398539	0.94194261	0.944530298
75th	0.952709981	0.947087723	0.946378373	0.947800194	0.949850244	0.942548951	0.944563468	0.948047308	0.948994771	0.94006913	0.942349763	0.943337504
76th	0.953702731	0.947951681	0.948398109	0.945271214	0.950354988	0.943436454	0.944566446	0.94828859	0.947693669	0.939852911	0.941890962	0.943273029
77th	0.95010903	0.945327483	0.946325828	0.947780441	0.951041324	0.945103267	0.947024912	0.94848	0.947958174	0.939655314	0.941677735	0.944456317
78th	0.952203372	0.946504543	0.946373234	0.94894302	0.948871571	0.938711981	0.940225792	0.947672987	0.948020573	0.939537706	0.9413906	0.944980689
79th	0.952725936	0.946531223	0.946767462	0.949421658	0.951561232	0.94411815	0.945485028	0.945268662	0.947520736	0.938707757	0.941001098	0.944248439
80th	0.951255965	0.94546122	0.945775867	0.949259529	0.950363687	0.94359122	0.944369787	0.947556923	0.94665509	0.938808163	0.94051734	0.944252931
81st	0.952486072	0.946730789	0.946862189	0.947589226	0.949445143	0.9416388	0.943747471	0.945581578	0.947124639	0.939238997	0.941240485	0.944878024
82nd	0.953293382	0.94732692	0.947668612	0.94962936	0.951844437	0.943761714	0.945582723	0.947960653	0.947507437	0.940195993	0.9421592	0.943514618
83rd	0.952600792	0.948010387	0.947774309	0.94575221	0.949461722	0.943512902	0.944185748	0.946644861	0.948255049	0.940061506	0.942809074	0.94467159
84th	0.952777632	0.946943817	0.947495664	0.949827304	0.951079997	0.943652352	0.945466576	0.947487713	0.947024459	0.93918432	0.941499236	0.944307032
85th	0.950069676	0.945547288	0.944653327	0.948184638	0.950361856	0.94363559	0.944295445	0.947294448	0.94775291	0.939742237	0.941732602	0.944645066
86th	0.950228743	0.944444444	0.946442657	0.948651689	0.95049668	0.944020636	0.944445969	0.948215759	0.94763986	0.94059222	0.942493444	0.944384771
87th	0.953251872	0.947523322	0.947129155	0.946669503	0.949322308	0.943078169	0.945177308	0.946675866	0.947758012	0.939595884	0.942077871	0.944099731
88th	0.951262447	0.947741783	0.943143901	0.946498054	0.950932339	0.943665424	0.94514651	0.94723943	0.948336783	0.939440443	0.942820943	0.944272212
89th	0.952970883	0.946921276	0.948867671	0.948911757	0.949331314	0.942399164	0.944105539	0.948070486	0.948164524	0.939774557	0.941510412	0.944306409
90th	0.952240613	0.946109518	NA	0.947826087	0.950226003	0.943445908	0.944746493	0.947622796	0.946665719	0.939790576	0.938407896	0.94478277
91st	0.951350075	0.947125351	0.944763704	0.947763006	0.950468983	0.942858666	0.945043701	0.948081514	0.947202746	0.939562692	0.941016379	0.943465852
92nd	0.950118203	0.944575781	0.945022327	0.948377491	0.95071862	0.944181838	0.945661663	0.948925756	0.947459045	0.939675199	0.94238096	0.945028975
93rd	0.953680837	0.947848248	0.948399979	0.948998179	0.949999467	0.942026668	0.943561539	0.948515485	0.947182752	0.939870687	0.942109632	0.945107593
94th	0.953176291	0.948134142	0.947582657	0.947643343	0.950231173	0.942575251	0.943621667	0.946963174	0.947534946	0.939534858	0.941808107	0.943960149
95th	0.952702525	0.947578632	0.947867672	0.945648205	0.94888121	0.942418037	0.942801988	0.947877876	0.948113569	0.940023396	0.942483246	0.943862252
96th	0.952796468	0.945305929	0.941889193	0.946810771	0.949966957	0.942964037	0.944989234	0.946912896	0.948306781	0.940510426	0.942527856	0.944802462
97th	0.95082484	0.946385416	0.945334664	0.949186004	0.948828725	0.9431675	0.94624147	0.946822261	0.947130619	0.939246924	0.940896873	0.944458754
98th	0.950304366	0.943875945	0.944190806	0.948415108	0.94990157	0.941367385	0.94396382	0.945050888	0.947812643	0.939585473	0.941541201	0.94506207
99th	0.956105697	0.949653235	0.950399438	0.946885166	0.950948067	0.943094636	0.944791233	0.946735649	0.948003648	0.939593357	0.941362795	0.944147147
100th	0.951373929	0.945436873	0.947013083	0.948373774	0.950495471	0.942802344	0.944453916	0.944786256	0.948364427	0.940697509	0.942473524	0.944585951
101st	0.951854279	0.945616564	0.944987551	0.947846977	0.950167035	0.943325542	0.944350165	0.947026068	0.94772593	0.939724574	0.942267725	0.94455642
102nd	0.951143315	0.946102754	0.946207766	0.949472211	0.952590015	0.945341838	0.947371328	0.946621147	0.947727285	0.939662196	0.941986035	0.943301562

103rd	0.951826544	0.944914586	0.945308804	0.947593615	0.951827384	0.945490374	0.947375883	0.946280127	0.947802813	0.939543903	0.941406527	0.944409131
104th	0.950912822	0.94667754	0.946440785	0.947028935	0.951826524	0.945781394	0.94621422	0.946803926	0.947713347	0.939618372	0.941602488	0.944417176
105th	0.951468039	0.947502495	0.947555019	0.948389447	0.953446281	0.946740646	0.948283536	0.947552534	0.948008125	0.940183681	0.941838116	0.944747664
106th	0.952907861	0.948057056	0.947191777	0.947125038	0.952873916	0.94724692	0.94986203	0.947972748	0.947776347	0.939872934	0.941635919	0.944928247
107th	0.949860724	0.944026909	0.945629894	0.947627438	0.95075927	0.948028769	0.945046542	0.9482502	0.947927731	0.939009034	0.942326855	0.944512683
108th	0.953779528	0.946692913	0.947427822	0.948427291	0.954556418	0.948058397	0.949633245	0.947053464	0.948512167	0.940869356	0.942821061	0.943716768
109th	0.951984887	0.945871487	0.945923963	0.952140291	0.94990249	0.948163587	0.945363422	0.946936664	0.947775491	0.940002947	0.94192425	0.944373152
110th	0.950213782	0.945020067	0.945649608	0.948187159	0.94993921	0.947312459	0.944638782	0.947895495	0.946837088	0.940088669	0.941655656	0.944988943
Mean	0.95180932	0.946218282	0.946355	0.9481544	0.950458319	0.943574845	0.944858969	0.947093095	0.947652395	0.939682438	0.9417369	0.944115396
SD	0.001152187	0.001332409	0.0013914	0.001320839	0.001089469	0.001665114	0.001621139	0.000982618	0.000535459	0.000556305	0.0006603	0.000606357

Note: two simulation failed due to technical issue and their concordance frequency are written as NA in the above table.

Table S5. Frequency of the markers that showed Mendelian inconsistency in Trios.

Population	Trios1 Markers frequency (No. of markers)	Trios2 Markers frequency (No. of markers)	Trios3 Markers frequency (No. of markers)	Trios4 Markers frequency (No. of markers)	Trios5 Markers frequency (No. of markers)
Vysya	0.0007 (607)	0.00106 (928)	0.00138 (1205)	0.00092 (799)	0.00105 (915)
Kashmiri Pandit	**0.10475 (91378)**	**0.17085 (149042)**	0.00066 (573)	0.00082 (711)	0.00154 (1345)
Mala	0.00074 (642)	0.00088 (764)	0.00112 (973)	0.00122 (1060)	0.0012 (1045)
Madiga	0.00074 (647)	0.00079 (686)	0.001 (870)	0.00098 (851)	0.00088 (766)
Kshatriya	0.00192 (1674)	0.00088 (766)	0.00078 (682)	0.00084 (729)	0.00073 (633)
Brahmin	0.00074 (645)	0.05494 (47930)	0.00073 (637)	0.00074 (642)	0.00117 (1024)

Note: The Trios1 and Trios2 of Kashmiri Pandit showed high rate of Mendelian inconsistency (>10%) and were removed from further analysis.

Table S6. List of Indian population samples used from our earlier study[1,2] for comparative analysis.

S. No	Population	Linguistic group	No. of individuals
1	Bhumij	Austro-Asiatic	5
2	Birhor	Austro-Asiatic	5
3	Ho	Austro-Asiatic	5
4	Kharia	Austro-Asiatic	5
5	Korku	Austro-Asiatic	5
6	Munda	Austro-Asiatic	5
7	Santhal	Austro-Asiatic	5
8	Adi-Dravider	Dravidian	5
9	Chenchu	Dravidian	7
10	Gond – Chattisgarh	Dravidian	5
11	Gond – Madhya Pradesh	Dravidian	5
12	Gond – Uttar Pradesh	Dravidian	5
13	Gounder	Dravidian	5
14	Hallaki	Dravidian	3
15	Irula	Dravidian	5
16	Jews Kerala	Dravidian	5
17	Kallar	Dravidian	5
18	Kamsali	Dravidian	5
19	Kattunayakan	Dravidian	5
20	Kurchian	Dravidian	5
21	Kurumba	Dravidian	6
22	Madiga	Dravidian	13
23	Mala	Dravidian	12
24	Malai Kuravar	Dravidian	5
25	Malli	Dravidian	5
26	Minicoy	Dravidian	5
27	Naidu	Dravidian	4
28	Narikkuravar	Dravidian	5
29	Palliyar	Dravidian	5
30	Paniyas	Dravidian	5
31	Vaish	Dravidian	2
32	Velama	Dravidian	3
33	Vysya	Dravidian	15
34	Bhil – Gujrat	Indo-European	11
35	Bhil – Madhya Pradesh	Indo-European	5
36	Brahmin	Indo-European	10
37	Jain	Indo-European	5
38	Kashmiri Pandit	Indo-European	15
39	Kshatriya – Rajasthan	Indo-European	5
40	Kshatriya – Uttar Pradesh	Indo-European	10
41	Lodi	Indo-European	5
42	Meghawal	Indo-European	4
43	Sahariya	Indo-European	3
44	Satnami	Indo-European	2
45	Srivastava	Indo-European	1
46	Tharu	Indo-European	4
47	Aonaga	Tibeto-Burman	4
48	Changkpa	Tibeto-Burman	5
49	Nyshi	Tibeto-Burman	3
50	Sherpa	Tibeto-Burman	5
51	Subba	Tibeto-Burman	5
52	Tibetan	Tibeto-Burman	5
53	Great Andamanese	Andamanese	5

Note: We used 150 Dravidians, 80 Indo-European, 35 Austro-Asiatic, 27 Tibeto-Burman and 5 Great Andamanese; and 15 trios of Dravidian (Vysya , Madiga, Mala; 5 each) and 15 trios of Indo-European (Kshatriya, Brahmin and Kashmiri Pandit; 5 each).

Table S7. Detection of outlier samples with sigma cutoff ±6 in 10 iteration on eigenvector 1-10.

S. No	Iteration number	Sample ID	Prakriti	Eigenvector	Sigma
1	1	P700	Pitta	1	8.084
2	1	P788	Pitta	8	-6.837
3	1	P774	Kapha	2	11.767
4	1	B034	Pitta	0	8.266
5	1	B046	Pitta	0	8.246
6	1	B052	Vata	0	8.377
7	1	B276	Kapha	9	6.476
8	1	B275	Kapha	9	6.476
9	1	B100	Pitta	4	6.977
10	1	B077	Vata	7	8.366
11	1	B047	Vata	7	8.366
12	1	U150	Kapha	9	6.119
13	1	P721	Kapha	3	8.274
14	1	U015	Kapha	9	6.064
15	1	P618	Kapha	5	-8.584
16	1	P654	Kapha	5	-8.582
17	1	P700	Pitta	1	8.144
18	1	P788	Pitta	8	-6.729
19	2	P1133	Vata	8	-9.111
20	2	P1040	Vata	0	6.628
21	2	P706	Pitta	1	9.471
22	2	P668	Kapha	4	-9.38
23	2	B282	Pitta	7	7.962
24	2	U076	Vata	2	7.64
25	2	P706	Pitta	1	10.042
26	2	B187	Pitta	2	7.488
27	2	B193	Vata	3	6.71
28	2	P1043	Kapha	0	6.678
29	2	P668	Kapha	4	-9.147
30	2	B161	Kapha	3	6.711
31	3	B283	Kapha	8	9.428
32	3	B255	Vata	6	-6.941
33	3	B103	Vata	5	-7.737
34	3	B123	Vata	9	-7.94
35	3	U012	Kapha	3	6.922
36	3	U113	Kapha	4	7.026
37	3	U120	Vata	9	6.689
38	4	B042	Kapha	7	-6.671
39	4	B059	Kapha	3	6.282
40	5	B140	Pitta	9	6.172

Note: After 5 iteration, no outlier samples were found.

Table S8. ANOVA statistics of *Prakriti* samples after outlier removal.

Eigenvector	Eigenvalue	*Prakriti I vs. Prakriti II*	ANOVA p-value
1	1.435535	Vata vs. Kapha	0.695176
		Vata vs. Pitta	0.426118
		Kapha vs. Pitta	0.2699
2	1.299304	Vata vs. Kapha	0.0311195
		Vata vs. Pitta	0.279802
		Kapha vs. Pitta	0.471693
3	1.236806	Vata vs. Kapha	0.440812
		Vata vs. Pitta	0.956564
		Kapha vs. Pitta	0.53893
4	1.150733	Vata vs. Kapha	0.859844
		Vata vs. Pitta	0.612884
		Kapha vs. Pitta	0.416545
5	1.12718	Vata vs. Kapha	0.38608
		Vata vs. Pitta	0.599178
		Kapha vs. Pitta	0.808545
6	1.120482	Vata vs. Kapha	0.900018
		Vata vs. Pitta	0.0536774
		Kapha vs. Pitta	0.0457074
7	1.112934	Vata vs. Kapha	0.513464
		Vata vs. Pitta	0.437969
		Kapha vs. Pitta	0.875392
8	1.106404	Vata vs. Kapha	0.879887
		Vata vs. Pitta	0.0819128
		Kapha vs. Pitta	0.0624001
9	1.102689	Vata vs. Kapha	0.213251
		Vata vs. Pitta	0.153697
		Kapha vs. Pitta	0.756238
10	1.096925	Vata vs. Kapha	0.146971
		Vata vs. Pitta	0.418338
		Kapha vs. Pitta	0.580848

Table S9. Association analysis and population structure as confounding factor.

SNP details			Eigenstrat				EMMAX		GCTA			
Chr	rsID	Physical Position	Armitage chisq	P-value	Eigenstrat chisq	P-value	BetaValue	P-value	Freq	Beta	SE	P-value
1	rs213490	54627572	18.197	1.99E-05	18.324	1.86E-05	-0.195586328	1.43E-05	0.421951	0.183966	0.0440512	2.96E-05
1	rs619413	59394979	18.127	2.07E-05	20.193	7.00E-06	0.194252368	1.46E-05	0.419512	-0.196835	0.0457044	1.66E-05
1	rs2269241	63881359	18.52	1.68E-05	18.096	2.10E-05	0.241382171	1.20E-05	0.207317	-0.231092	0.0561943	3.92E-05
1	rs2269240	63881852	17.892	2.34E-05	17.435	2.97E-05	0.237576989	1.71E-05	0.204878	-0.227571	0.056277	5.26E-05
1	rs2269239	63881947	17.892	2.34E-05	17.435	2.97E-05	0.237576989	1.71E-05	0.204878	-0.227571	0.056277	5.26E-05
1	rs2269238	63890125	17.275	3.23E-05	17.106	3.54E-05	0.234035256	2.38E-05	0.202439	-0.223742	0.0563748	7.22E-05
1	rs6688707	100040996	17.901	2.33E-05	19.051	1.27E-05	0.193101915	1.70E-05	0.439024	-0.196718	0.0438503	7.25E-06
1	rs11166345	100045548	17.901	2.33E-05	19.051	1.27E-05	0.193101915	1.70E-05	0.439024	-0.196718	0.0438503	7.25E-06
1	rs6586471	104648021	20.733	5.28E-06	20.43	6.19E-06	-0.229329208	3.37E-06	0.378049	0.221177	0.0485528	5.23E-06
2	rs10197747	57644785	18.162	2.03E-05	17.377	3.06E-05	-0.644329991	1.46E-05	0.0268293	0.620156	0.145749	2.09E-05
2	rs13396046	57707740	18.162	2.03E-05	17.377	3.06E-05	-0.644329991	1.46E-05	0.0268293	0.620156	0.145749	2.09E-05
2	rs6748662	57710603	18.162	2.03E-05	17.377	3.06E-05	-0.644329991	1.46E-05	0.0268293	0.620156	0.145749	2.09E-05
2	rs1376616	79479938	17.969	2.25E-05	20.342	6.48E-06	-0.498019348	1.63E-05	0.0463415	0.523608	0.113258	3.78E-06
2	rs11902715	164190114	18.288	1.90E-05	18.474	1.72E-05	-0.322637321	1.36E-05	0.121951	0.321006	0.0727299	1.02E-05
2	rs1440472	164193843	17.755	2.51E-05	18.436	1.76E-05	-0.280297715	1.84E-05	0.168293	0.2855	0.064126	8.50E-06
2	rs6731290	164196489	17.44	2.97E-05	17.859	2.38E-05	-0.339615215	2.20E-05	0.104878	0.335904	0.0783958	1.83E-05
2	rs13429987	184201539	16.506	4.85E-05	13.318	0.0002629	0.215179401	3.74E-05	0.258537	-0.204938	0.0508869	5.64E-05
2	rs986846	239172751	17.476	2.91E-05	16.873	4.00E-05	-0.225696305	3.91E-06	0.446341	0.225294	0.0480849	2.79E-06
2	rs986846	239172751	20.474	6.05E-06	20.314	6.57E-06	0.198918743	2.16E-05	0.446341	-0.19314	0.0457161	2.39E-05
3	rs7616568	2045846	18.387	1.80E-05	14.967	0.00010941	0.198589056	1.29E-05	0.492683	-0.184111	0.044496	3.51E-05
3	rs9815240	147394867	20.044	7.57E-06	17.733	2.54E-05	-0.401704714	5.00E-06	0.0707317	0.367374	0.0862046	2.03E-05
3	rs9830734	186033195	20.263	6.75E-06	18.591	1.62E-05	-0.227749903	4.41E-06	0.492683	0.21413	0.0487739	1.13E-05
3	rs10446349	186131720	17.278	3.23E-05	17.169	3.42E-05	-0.210703848	2.42E-05	0.478049	0.209463	0.0488663	1.82E-05
4	rs2939743	37219957	19.263	1.14E-05	18.929	1.36E-05	0.187726761	7.81E-06	0.417073	-0.182453	0.0410944	9.00E-06
5	rs420150	141164192	19.833	8.45E-06	16.766	4.23E-05	-0.28183339	5.80E-06	0.153659	0.250716	0.0635643	8.00E-05
5	rs10866665	172908297	17.375	3.07E-05	16.842	4.06E-05	0.197103111	2.29E-05	0.456098	-0.196801	0.0455844	1.58E-05
5	rs7717957	172914029	17.375	3.07E-05	16.812	4.13E-05	0.197103111	2.29E-05	0.456098	-0.195957	0.0455844	1.72E-05
6	rs2655668	80248839	17.686	2.61E-05	13.699	0.00021456	-0.199745656	1.99E-05	0.297561	0.173086	0.0476819	0.00028341
6	rs767789	80290087	17.085	3.58E-05	13.48	0.00024116	-0.183853673	2.81E-05	0.431707	0.159486	0.0446507	0.000354462
6	rs790607	91163700	16.745	4.27E-05	15.457	8.44E-05	0.18619412	3.35E-05	0.463415	-0.16762	0.0457178	0.000245978
6	rs12526892	163641063	19.557	9.76E-06	18.448	1.75E-05	0.245880892	6.61E-06	0.268293	-0.23655	0.0535982	1.02E-05

7	rs10231685	103963525	19.156	1.20E-05	13.38	0.0002543	-0.259623046	8.94E-06	0.173171	0.207816	0.0596195	0.000490825
7	rs6969323	104001723	17.683	2.61E-05	12.427	0.00042326	-0.23247942	2.02E-05	0.202439	0.195097	0.0555266	0.000442118
10	rs17731	3811561	22.692	1.90E-06	22.576	2.02E-06	-0.232364982	1.09E-06	0.321951	0.215453	0.0468657	4.28E-06
10	rs2398215	10425666	20.524	5.89E-06	21.916	2.85E-06	0.224125475	3.92E-06	0.42439	-0.231227	0.0496103	3.15E-06
10	rs10994948	63270054	19.256	1.14E-05	17.281	3.22E-05	-0.213094539	7.61E-06	0.343902	0.203748	0.0485908	2.75E-05
10	rs2901714	95399009	17.577	2.76E-05	13.994	0.00018343	-0.201746885	2.04E-05	0.482927	0.180935	0.0463887	9.60E-05
10	rs6583901	95399988	17.76	2.51E-05	15.087	0.00010265	-0.225907191	1.84E-05	0.226829	0.211845	0.0516756	4.14E-05
10	rs1326217	95400989	17.589	2.74E-05	14.711	0.00012532	-0.199767451	2.02E-05	0.487805	0.185931	0.0459181	5.14E-05
10	rs12781996	108025031	17.357	3.10E-05	13.236	0.00027461	0.264593992	2.31E-05	0.197561	-0.231126	0.0612235	0.000159932
11	rs1439528	96115783	18.657	1.56E-05	16.834	4.08E-05	-0.209702416	1.10E-05	0.492683	0.198192	0.0468016	2.29E-05
12	rs3759173	113607258	17.964	2.25E-05	21.786	3.05E-06	-0.37130311	1.64E-05	0.0780488	0.392915	0.0844523	3.28E-06
12	rs10848194	129791290	18.445	1.75E-05	18.571	1.64E-05	0.197531472	1.25E-05	0.417073	-0.195933	0.0441896	9.25E-06
14	rs1862127	77477049	20.879	4.89E-06	19.79	8.65E-06	-0.27020634	3.10E-06	0.209756	0.250679	0.0568152	1.02E-05
14	rs4903671	77481783	21.023	4.54E-06	19.273	1.13E-05	-0.273675684	2.86E-06	0.195122	0.252691	0.0573462	1.05E-05
14	rs4020132	77481947	20.879	4.89E-06	19.79	8.65E-06	-0.27020634	3.10E-06	0.209756	0.250679	0.0568152	1.02E-05
15	rs1664454	55469925	21.34	3.85E-06	19.612	9.49E-06	-0.225562079	2.38E-06	0.378049	0.212703	0.0470707	6.22E-06
15	rs10518915	55472117	17.878	2.36E-05	16.023	6.26E-05	-0.219953077	2.69E-06	0.463415	0.209321	0.0461332	5.70E-06
15	rs10518915	55472117	21.125	4.30E-06	19.849	8.38E-06	0.188387771	1.78E-05	0.463415	-0.173009	0.0447335	0.000109939
15	rs2892383	91093506	18.218	1.97E-05	17.272	3.24E-05	0.183961194	1.43E-05	0.470732	-0.176989	0.0431886	4.17E-05
15	rs2388017	91094840	17.357	3.10E-05	16.498	4.87E-05	0.179381998	2.31E-05	0.458537	-0.173149	0.0431282	5.95E-05
17	rs4789092	70203226	20.53	5.87E-06	16.632	4.54E-05	-0.367378973	3.86E-06	0.095122	0.311824	0.0815995	0.000132695
19	rs8103262	57757626	17.614	2.71E-05	16.993	3.75E-05	-0.24549101	2.00E-05	0.180488	0.240333	0.0561988	1.90E-05
20	rs6039614	9815136	16.983	3.77E-05	15.853	6.85E-05	0.189897608	2.85E-05	0.495122	-0.191366	0.0442719	1.54E-05

Table S10. The list of 52 markers and their frequency distribution among the three centres.

Markers	Chr	rsID	Allele-I	Allele-II	Allele-I frequency			Chisq	P-value
					Bangalore (B) 40 individuals	Pune (P) 75 individuals	Udupi (U) 90 individuals		
1	1	rs213490	T	C	0.4125	0.38	0.4611	2.2433	0.3257
2	1	rs619413	T	G	0.4125	0.4133	0.4278	0.0902	0.9559
3	1	rs2269241	G	A	0.2	0.2733	0.1556	6.9386	0.03114
4	1	rs2269240	G	A	0.2	0.2733	0.15	7.6543	0.02177
5	1	rs2269239	C	G	0.2	0.2733	0.15	7.6543	0.02177
6	1	rs6688707	T	G	0.4125	0.44	0.45	0.3172	0.8534
7	1	rs11166345	A	G	0.4125	0.44	0.45	0.3172	0.8534
8	1	rs6586471	T	C	0.4	0.4267	0.3278	3.6065	0.1648
9	1	rs2269238	A	C	0.1611	0.26	0.1875	5.6753	0.05856
10	2	rs10197747	C	A	0.025	0.04	0.01667	1.7188	0.4234
11	2	rs13396046	A	G	0.025	0.04	0.01667	1.7188	0.4234
12	2	rs6748662	G	A	0.025	0.04	0.01667	1.7188	0.4234
13	2	rs11902715	G	A	0.1125	0.1533	0.1	2.2563	0.3236
14	2	rs986846	T	C	0.425	0.48	0.4278	1.0861	0.581
15	2	rs1376616	C	T	0.03333	0.06	0.05	2.1119	0.3479
16	2	rs1440472	T	C	0.1667	0.1867	0.1375	0.9071	0.6354
17	2	rs6731290	C	T	0.07778	0.14	0.1	3.3994	0.1827
18	2	rs13429987	G	A	0.2444	0.2533	0.3	1.1536	0.5617
19	3	rs7616568	G	A	0.4875	0.5	0.4889	0.0511	0.9748
20	3	rs9815240	C	A	0.0375	0.1133	0.05	6.6629	0.03574
21	3	rs9830734	T	C	0.375	0.5	0.5389	6.0024	0.04973
22	3	rs10446349	G	A	0.5056	0.4933	0.3875	3.2109	0.2008
23	4	rs2939743	A	G	0.425	0.4533	0.3833	1.6747	0.4329
24	5	rs420150	A	C	0.2375	0.1333	0.1333	5.3725	0.06814
25	5	rs10866665	G	A	0.5375	0.4333	0.4389	2.6651	0.2638
26	5	rs7717957	C	G	0.55	0.4267	0.4389	3.5822	0.1668
27	6	rs2655668	C	T	0.3125	0.2667	0.3167	1.0847	0.5814
28	6	rs767789	T	C	0.425	0.44	0.4278	0.068	0.9666
29	6	rs12526892	A	G	0.3125	0.2133	0.2944	3.7314	0.1548
30	6	rs790607	A	G	0.4667	0.4667	0.45	0.0719	0.9647
31	7	rs10231685	G	A	0.2	0.1733	0.1611	0.585	0.7464
32	7	rs6969323	A	C	0.275	0.18	0.1889	3.2813	0.1939
33	10	rs17731	A	G	0.325	0.3533	0.2944	1.304	0.521
34	10	rs2398215	A	G	0.425	0.44	0.4111	0.2797	0.8695
35	10	rs10994948	G	A	0.3875	0.32	0.3444	1.054	0.5904
36	10	rs2901714	A	T	0.425	0.4867	0.5056	1.4525	0.4837
37	10	rs6583901	T	C	0.25	0.22	0.2222	0.3066	0.8579
38	10	rs1326217	T	A	0.4375	0.4933	0.5056	1.0556	0.5899
39	10	rs12781996	A	G	0.1944	0.1867	0.225	0.5675	0.7529
40	11	rs1439528	A	G	0.475	0.5267	0.4722	1.0947	0.5785
41	12	rs3759173	A	C	0.0625	0.08667	0.07778	0.4238	0.8091
42	12	rs10848194	A	C	0.325	0.4133	0.4611	4.234	0.1204
43	14	rs1862127	T	C	0.2	0.22	0.2056	0.1601	0.9231
44	14	rs4903671	A	C	0.2	0.1933	0.1944	0.0157	0.9922
45	14	rs4020132	A	G	0.2	0.22	0.2056	0.1601	0.9231
46	15	rs1664454	T	C	0.4125	0.44	0.3111	6.2824	0.04323
47	15	rs10518915	C	A	0.4375	0.5133	0.4333	2.3743	0.3051
48	15	rs2892383	T	C	0.4875	0.44	0.4889	0.8971	0.6386
49	15	rs2388017	T	C	0.4625	0.4267	0.4833	1.0645	0.5873
50	17	rs4789092	C	A	0.15	0.04667	0.1111	7.4254	0.02441
51	19	rs8103262	C	T	0.2375	0.1333	0.1944	4.25	0.1194
52	20	rs6039614	C	A	0.525	0.5	0.4778	0.5166	0.7724

Note: Cut of bonferroni adjustment without correction p-value for first 52 marker is 9.6×10^{-4}

Table S11. Prediction of *Prakrities* in 205 Ayurvedic samples with proposed statistical model.

Sample ID	W_{vs}	W_{ps}	W_{ks}	V *VP*	V *VK*	V *PK*	V *PV*	V *KP*	V *KV*	R_V	R_P	R_K	Predicted *Prakriti*	True *Prakriti*
B053	-1.291	-1.247	1.750	-0.044	-3.041	-2.997	0.044	2.997	3.041	3.041	2.997	4.269	K	K
B074	-0.737	1.616	-1.074	-2.353	0.337	2.690	2.353	-2.690	-0.337	2.377	3.574	2.711	P	P
B085	-0.627	1.926	-0.515	-2.553	-0.112	2.441	2.553	-2.441	0.112	2.556	3.533	2.444	P	P
B107	1.825	-0.059	-1.124	1.884	2.949	1.065	-1.884	-1.065	-2.949	3.500	2.164	3.136	V	V
B115	-0.291	1.118	-1.556	-1.409	1.265	2.674	1.409	-2.674	-1.265	1.894	3.023	2.958	P	P
B126	1.224	-1.096	-0.766	2.320	1.990	-0.330	-2.320	0.330	-1.990	3.056	2.343	2.017	V	V
B128	-1.035	0.297	1.732	-1.332	-2.766	-1.435	1.332	1.435	2.766	3.070	1.957	3.116	K	K
B148	-1.323	0.881	-1.752	-2.204	0.429	2.633	2.204	-2.633	-0.429	2.245	3.434	2.668	P	P
B156	-0.207	1.568	-1.896	-1.775	1.689	3.464	1.775	-3.464	-1.689	2.450	3.892	3.854	P	P
B157	0.253	-1.690	1.390	1.943	-1.137	-3.080	-1.943	3.080	1.137	2.251	3.642	3.283	K	K
B205	-1.115	0.075	1.816	-1.190	-2.931	-1.741	1.190	1.741	2.931	3.163	2.109	3.409	K	K
B206	-0.694	-0.078	2.058	-0.616	-2.753	-2.136	0.616	2.136	2.753	2.821	2.223	3.484	K	K
B210	-0.279	-0.949	2.115	0.671	-2.394	-3.065	-0.671	3.065	2.394	2.486	3.137	3.889	K	K
B254	2.689	-0.872	0.338	3.562	2.352	-1.210	-3.562	1.210	-2.352	4.268	3.762	2.645	V	V
B260	-0.511	1.368	2.433	-1.880	-2.944	-1.064	1.880	1.064	2.944	3.493	2.160	3.130	K	K
B271	-0.492	-0.442	1.898	-0.050	-2.390	-2.340	0.050	2.340	2.390	2.391	2.341	3.345	K	K
B277	-0.854	1.599	-0.563	-2.453	-0.290	2.162	2.453	-2.162	0.290	2.470	3.270	2.182	P	P
B288	-1.691	-0.126	1.549	-1.566	-3.240	-1.675	1.566	1.675	3.240	3.599	2.293	3.648	K	K
P1049	1.772	-1.840	-0.494	3.612	2.267	-1.346	-3.612	1.346	-2.267	4.265	3.855	2.636	V	V
P1053	-0.407	-1.234	1.483	0.827	-1.890	-2.717	-0.827	2.717	1.890	2.063	2.840	3.309	K	K
P1055	-1.095	0.068	1.635	-1.163	-2.730	-1.567	1.163	1.567	2.730	2.967	1.951	3.147	K	K
P1073	1.457	-1.853	-1.637	3.310	3.094	-0.217	-3.310	0.217	-3.094	4.531	3.317	3.101	V	V
P1111	-1.159	-1.100	1.767	-0.060	-2.926	-2.867	0.060	2.867	2.926	2.927	2.867	4.096	K	K
P1114	0.996	-1.373	-0.919	2.369	1.914	-0.454	-2.369	0.454	-1.914	3.046	2.412	1.968	V	V
P1164	2.111	-0.761	-0.532	2.872	2.643	-0.229	-2.872	0.229	-2.643	3.904	2.881	2.653	V	V
P1183	-1.061	-0.899	1.272	-0.163	-2.333	-2.170	0.163	2.170	2.333	2.339	2.177	3.186	K	K
P1234	-1.088	1.255	-1.386	-2.343	0.298	2.641	2.343	-2.641	-0.298	2.362	3.530	2.657	P	P
P766	-0.667	2.005	-0.144	-2.672	-0.523	2.150	2.672	-2.150	0.523	2.723	3.430	2.212	P	P
P1048	2.520	-0.283	-1.377	2.802	3.897	1.094	-2.802	-1.094	-3.897	4.800	3.009	4.048	V	V
P1101	2.383	-0.113	-0.411	2.496	2.794	0.298	-2.496	-0.298	-2.794	3.746	2.513	2.810	V	V
P1107	2.031	-1.198	-2.114	3.229	4.146	0.916	-3.229	-0.916	-4.146	5.255	3.357	4.246	V	V
P055	1.860	-0.187	-0.780	2.046	2.640	0.594	-2.046	-0.594	-2.640	3.340	2.131	2.706	V	V
P542	-1.557	-1.207	2.447	-0.351	-4.004	-3.654	0.351	3.654	4.004	4.020	3.670	5.421	K	K
P606	1.884	-0.838	-0.460	2.721	2.343	-0.378	-2.721	0.378	-2.343	3.591	2.748	2.374	V	V
P835	0.113	1.320	-1.480	-1.206	1.594	2.800	1.206	-2.800	-1.594	1.999	3.049	3.222	P	P
P848	-0.127	-0.829	1.816	0.702	-1.943	-2.645	-0.702	2.645	1.943	2.066	2.737	3.282	K	K
U020	-0.919	-1.370	1.952	0.451	-2.871	-3.322	-0.451	3.322	2.871	2.906	3.353	4.391	K	K
U040	-1.037	-1.395	1.906	0.358	-2.944	-3.301	-0.358	3.301	2.944	2.965	3.320	4.423	K	K
U049	0.676	-1.711	-1.419	2.387	2.095	-0.292	-2.387	0.292	-2.095	3.176	2.404	2.115	V	V
U071	3.013	-0.868	0.288	3.881	2.724	-1.156	-3.881	1.156	-2.724	4.742	4.049	2.960	V	V
U078	-0.989	0.293	2.408	-1.282	-3.397	-2.115	1.282	2.115	3.397	3.631	2.474	4.002	K	K
U104	-0.639	-1.352	2.201	0.713	-2.841	-3.554	-0.713	3.554	2.841	2.929	3.624	4.549	K	K
U146	2.720	-1.345	-0.327	4.066	3.047	-1.019	-4.066	1.019	-3.047	5.081	4.191	3.213	V	V
U165	1.523	-1.564	-0.988	3.087	2.511	-0.576	-3.087	0.576	-2.511	3.980	3.141	2.577	V	V
U168	-0.975	-1.668	0.923	0.693	-1.897	-2.591	-0.693	2.591	1.897	2.020	2.682	3.211	K	K
U181	-0.499	1.609	-0.833	-2.108	0.335	2.442	2.108	-2.442	-0.335	2.134	3.226	2.465	P	P
U194	-0.446	2.171	-1.046	-2.617	0.600	3.217	2.617	-3.217	-0.600	2.685	4.147	3.272	P	P
U226	-2.145	2.074	-1.413	-4.219	-0.732	3.487	4.219	-3.487	0.732	4.282	5.473	3.563	P	P
U315	-0.785	1.909	-1.429	-2.694	0.644	3.338	2.694	-3.338	-0.644	2.770	4.290	3.400	P	P

Table S12. The prediction of *Prakrities* in Indian population samples with proposed statistical model.

Sample-ID	Ancestry	W_{vs}	W_{ps}	W_{ks}	V *KP*	V *KV*	V *PK*	V *PV*	V *VP*	V *VK*	R_K	R_P	R_V	Predicted *Prakriti*
Bhil_MP02	Austro-Asiatic	-2.398	2.076	-1.591	-3.667	0.807	3.667	4.474	-4.474	-0.807	3.755	5.784	4.546	P
Bhil_MP03	Austro-Asiatic	0.262	1.383	-1.592	-2.976	-1.855	2.976	1.121	-1.121	1.855	3.506	3.180	2.167	P
Bhil97	Indo-European	-1.424	1.223	-0.799	-2.022	0.625	2.022	2.646	-2.646	-0.625	2.116	3.330	2.719	P
Bhumihar04	Austro-Asiatic	-0.990	-2.914	0.456	3.370	1.446	-3.370	-1.924	1.924	-1.446	3.667	3.880	2.407	K
Bhumihar05	Austro-Asiatic	-0.658	1.054	-1.462	-2.516	-0.804	2.516	1.712	-1.712	0.804	2.641	3.043	1.891	P
Brahmin1F	Indo-European	-1.770	-0.037	1.178	1.215	2.948	-1.215	1.733	-1.733	-2.948	3.189	2.117	3.420	K
Brahmin3F	Indo-European	-2.338	0.938	2.152	1.214	4.490	-1.214	3.276	-3.276	-4.490	4.651	3.494	5.558	K
Changpa05	Tibeto-Burman	-0.403	0.765	-2.702	-3.467	-2.299	3.467	1.168	-1.168	2.299	4.160	3.659	2.579	P
Chenchu31	Dravidian	-2.316	0.504	-1.138	-1.642	1.178	1.642	2.820	-2.820	-1.178	2.021	3.263	3.056	P
Chenchu37	Dravidian	0.074	1.640	-1.289	-2.929	-1.364	2.929	1.565	-1.565	1.364	3.231	3.321	2.076	P
Gond_MP05	Dravidian	-1.063	-2.019	1.542	3.560	2.604	-3.560	-0.956	0.956	-2.604	4.411	3.686	2.774	K
Jews04	Dravidian	-2.475	-1.846	2.592	4.438	5.067	-4.438	0.629	-0.629	-5.067	6.736	4.483	5.106	K
Kall01	Dravidian	0.219	2.346	-0.423	-2.769	-0.642	2.769	2.127	-2.127	0.642	2.842	3.492	2.222	P
Kashmiri_pandit2F	Indo-European	-0.737	1.490	-2.274	-3.764	-1.537	3.764	2.227	-2.227	1.537	4.065	4.373	2.706	P
Kashmiri_pandit2M	Indo-European	0.961	-1.175	-1.973	-0.798	-2.934	0.798	-2.136	2.136	2.934	3.040	2.280	3.629	V
Kshatriya_Rj5F	Indo-European	-0.928	1.505	-1.028	-2.533	-0.100	2.533	2.433	-2.433	0.100	2.535	3.512	2.435	P
Kshatriya_UP5F	Indo-European	-0.975	2.101	-0.482	-2.584	0.493	2.584	3.077	-3.077	-0.493	2.630	4.018	3.116	P
Kshatriya_UP5M	Indo-European	1.406	-1.608	-0.636	0.972	-2.041	-0.972	-3.014	3.014	2.041	2.261	3.167	3.640	V
Kurumba46	Dravidian	-0.405	-1.990	1.596	3.585	2.001	-3.585	-1.584	1.584	-2.001	4.106	3.920	2.552	K
Lodi05	Dravidian	-0.284	-0.063	2.106	2.169	2.391	-2.169	0.222	-0.222	-2.391	3.228	2.180	2.401	K
Madiga3F	Dravidian	-1.489	0.904	-1.808	-2.712	-0.319	2.712	2.393	-2.393	0.319	2.731	3.617	2.414	P
Madiga4F	Dravidian	-1.193	1.838	-0.390	-2.228	0.803	2.228	3.032	-3.032	-0.803	2.369	3.762	3.136	P
Mala5M	Dravidian	-0.898	-1.644	0.950	2.594	1.849	-2.594	-0.745	0.745	-1.849	3.186	2.699	1.993	K
Minicoy01	Dravidian	-1.459	-1.053	2.046	3.098	3.504	-3.098	0.406	-0.406	-3.504	4.678	3.125	3.528	K
Munda01	Dravidian	1.209	-1.771	-0.524	1.246	-1.734	-1.246	-2.980	2.980	1.734	2.135	3.230	3.447	V
Munda02	Dravidian	-1.766	1.164	-1.655	-2.819	0.111	2.819	2.929	-2.929	-0.111	2.821	4.065	2.931	P
Naidu77	Dravidian	0.004	-1.482	-2.983	-1.501	-2.987	1.501	-1.486	1.486	2.987	3.343	2.112	3.337	V
Naidu80	Dravidian	-0.542	1.835	-1.130	-2.965	-0.588	2.965	2.377	-2.377	0.588	3.023	3.800	2.449	P
Narikuravar04	Dravidian	2.781	0.758	-0.514	-1.272	-3.295	1.272	-2.023	2.023	3.295	3.532	2.389	3.866	V
Paliyar04	Dravidian	-1.151	1.282	-0.873	-2.155	0.278	2.155	2.434	-2.434	-0.278	2.173	3.251	2.449	P
Paniyan01	Dravidian	-1.032	-1.349	1.168	2.517	2.199	-2.517	-0.317	0.317	-2.199	3.342	2.537	2.222	K
Paniyan02	Dravidian	-0.492	1.977	-1.131	-3.108	-0.639	3.108	2.469	-2.469	0.639	3.173	3.970	2.550	P
Santhal77	Austro-Asiatic	0.083	-1.098	-2.719	-1.621	-2.802	1.621	-1.181	1.181	2.802	3.237	2.006	3.041	V
Subba01	Tibeto-Burman	-1.583	0.749	1.823	1.074	3.406	-1.074	2.332	-2.332	-3.406	3.571	2.567	4.127	K
Velama83	Dravidian	2.365	-1.175	0.114	1.290	-2.251	-1.290	-3.541	3.541	2.251	2.594	3.768	4.196	V
Vysya2F	Dravidian	-0.498	2.076	0.391	-1.685	0.889	1.685	2.574	-2.574	-0.889	1.905	3.077	2.723	P
Vysya53	Dravidian	-1.824	1.371	0.361	-1.011	2.185	1.011	3.195	-3.195	-2.185	2.407	3.351	3.871	P

Table S13. The list of variants of Kapha and Pitta individuals present in *PGM1* gene.

Sample ID	Prakriti	Physical position: hg19																						
		64059644	64059863	64089405	64095111	64097432	64100478	64100693	64102058	64104302	64104307	64114301	64114303	64117205	64117319	64117414	64117537	64120149	64125234	64125368	64125439	64125544	64125793	64125944
		Wild type > Mutant allele																						
		C>G	T>A	A>G	A>G	C>T	A>T	G>T	C>T	A>G	C>T	T>C	T>C	T>delT	C>T	G>A	G>T	C>T	G>T	C>T	A>C	C>T	G>A	C>G
B014	Kapha	GG	-	-	-	TT	TT	-	-	-	TC	-	-	-	-	-	-	-	-	-	-	-	-	-
B016	Kapha	-	-	-	-	-	-	-	-	-	-	CT	-	-	-	-	TG	-	TG	TC	CA	-	-	-
B018	Kapha	GG	-	-	-	-	-	-	-	-	-	-	-	-	-	-	-	-	-	-	-	-	-	-
B020	Kapha	GG	AT	-	-	TC	TA	TG	-	-	-	-	-	-	-	-	-	-	-	-	-	-	-	-
B024	Pitta	GG	-	-	-	TC	TA	-	-	-	-	CT	-	-	-	-	TG	-	TG	TC	CA	-	-	-
B041	Pitta	GC	AT	-	-	-	-	-	-	-	-	-	-	-	-	-	-	-	-	-	-	-	-	-
B048	Pitta	GC	-	-	-	TC	TA	-	-	-	-	-	-	-	-	-	-	-	-	-	-	TC	-	-
B051	Kapha	GG	-	-	-	TC	TA	-	-	-	-	-	-	-	-	-	-	-	-	-	-	-	-	-
B053	Kapha	GC	-	-	GA	-	TA	TG	-	-	-	CT	-	-	-	-	TG	-	TT	TC	CA	-	-	-
B055	Pitta	GG	-	-	-	-	-	-	-	-	-	-	-	-	-	-	-	-	-	-	-	-	-	-
B060	Pitta	GG	AT	-	-	TC	TA	-	-	-	-	-	CT	-	-	-	-	-	TG	-	-	-	-	-
B061	Pitta	-	-	-	-	TC	TA	-	-	-	-	CT	-	-	-	-	TG	-	TG	TC	CA	TC	-	-
B070	Pitta	GC	AT	-	-	-	-	-	-	-	-	-	-	-	-	-	-	-	-	-	-	-	-	-
B073	Pitta	-	-	-	-	TC	TT	-	-	-	-	-	-	-	-	-	-	-	TG	-	-	-	-	-
B074	Pitta	GC	AT	-	-	-	TT	-	-	-	-	-	-	-	-	-	-	-	-	-	-	TC	-	-
B076	Pitta	GC	-	-	-	-	TA	-	-	-	-	-	-	-	-	-	-	-	-	-	-	-	-	-
B083	Pitta	-	-	-	-	TC	TA	-	-	-	-	-	-	-	-	-	-	-	-	-	-	-	-	-
B097	Pitta	GG	-	-	-	-	-	-	-	-	-	-	-	-	-	-	-	-	TG	-	-	-	-	-
B102	Pitta	GG	-	-	-	TC	TA	-	-	-	-	-	-	-	-	-	-	-	-	-	-	-	-	-
B115	Pitta	GG	-	-	-	-	-	-	-	-	-	-	-	-	-	-	-	-	-	-	-	TC	-	-
B121	Pitta	GG	-	-	-	TC	TA	-	TC	-	-	-	-	-	-	-	-	-	-	-	-	-	-	-
B128	Kapha	GC	-	-	-	TT	TT	TG	-	-	TC	-	-	-	-	-	-	-	-	-	-	-	-	-
B135	Pitta	GC	-	-	-	-	-	-	-	-	-	-	-	-	-	-	-	-	-	-	-	-	-	-
B147	Pitta	-	-	-	-	TC	TA	-	-	-	-	-	-	-	-	-	-	-	-	-	-	-	-	-
B149	Pitta	GC	-	-	-	TC	TT	-	-	-	TC	-	-	-	-	-	-	-	-	-	-	TC	-	GC
B156	Pitta	GC	-	-	-	TC	TT	-	-	-	-	-	-	-	-	-	-	-	-	-	-	-	-	-
B162	Pitta	GG	AT	-	-	TC	TA	-	-	-	-	-	-	-	-	-	-	-	TG	-	-	-	-	-
B166	Pitta	GG	-	-	GA	TC	TA	-	-	-	-	-	-	-T	-	-	-	-	-	-	-	-	-	-
B169	Pitta	GG	AA	-	-	-	TA	-	-	-	-	-	-	-	-	-	-	-	-	-	-	-	-	-
B199	Pitta	GG	-	-	-	TC	TT	-	-	-	TC	-	-	-	-	-	-	-	-	-	-	TC	AG	-
B205	Kapha	GG	-	-	-	TC	TT	-	-	-	-	-	-	-	-	-	-	TC	-	-	-	-	-	-
B206	Kapha	-	-	-	-	TT	TT	TG	-	-	-	-	-	-	-	-	-	-	-	-	-	-	-	-

B247	Kapha	GC	-	-	GA	-	TA	-	-	-	-	-	-	-	-	-	-	-	-	-	-	-	-	-
B261	Kapha	GG	AT	-	GA	TC	TA	TG	-	-	-	CT	-	-	-	-	TG	-	TG	TC	CA	-	-	-
B263	Kapha	GC	-	-	GA	TC	TA	TG	-	-	-	CT	-	-	-	-	TG	-	TG	TC	CA	-	-	-
B277	Pitta	GG	-	-	-	TC	TA	-	-	-	-	-	-	-	-	-	-	-	TG	-	-	TC	-	-
B280	Kapha	GG	-	GA	-	TC	TA	-	-	-	-	-	-	-	-	-	-	-	TG	-	-	-	-	-
B281	Kapha	GC	-	-	-	-	TA	-	-	-	-	-	-	-	-	-	-	-	-	-	-	-	-	-
B285	Kapha	GG	-	-	GA	TT	TT	TG	-	-	TC	CT	-	-	-	-	TG	-	TG	TC	CA	TC	-	-
B288	Kapha	GG	-	-	-	TC	TA	-	-	-	-	-	-	-	-	-	-	-	-	-	-	-	-	-
B289	Kapha	GG	-	-	GA	-	-	TG	-	-	-	-	-	-	-	-	-	-	-	-	-	-	-	-
P018	Kapha	GC	AT	-	-	TT	TT	TG	-	-	-	-	-	-	-	-	-	-	-	-	-	TC	-	-
P1045	Kapha	GG	AT	-	-	TC	TA	-	-	-	-	-	-	-	-	-	-	-	-	-	-	-	-	-
P1053	Kapha	GG	-	-	-	TC	TT	-	-	-	TC	CT	-	-	-	-	TG	-	TG	TC	CA	-	-	-
P1055	Kapha	GG	-	-	-	TC	TA	TG	-	-	-	-	-	-	-	-	-	-	-	-	-	-	-	-
P1087	Kapha	GG	-	-	-	-	TA	-	-	-	-	-	-	-	TC	-	-	-	-	-	-	-	-	-
P1096	Kapha	GG	AT	-	-	-	-	-	-	-	-	CT	-	-	-	-	TG	-	TG	TC	CA	-	-	-
P1099	Kapha	GG	AA	-	-	TC	TA	TG	-	GA	TC	-	-	-	-	-	-	-	-	-	-	-	-	-
P1111	Kapha	GC	-	-	-	-	-	TG	-	-	-	CT	-	-	-	-	TG	-	TG	TC	CA	-	-	-
P1115	Kapha	GG	-	-	-	TC	TT	TG	-	-	-	CT	-	-	-	-	TG	-	TG	TC	CA	-	-	-
P1191	Pitta	GC	-	-	GA	TT	TT	-	-	-	-	-	-	-	-	-	-	-	-	-	-	-	-	-
P1210	Kapha	GG	-	-	-	TC	TA	TG	-	-	-	CT	-	-	-	-	TG	-	-	-	-	-	-	-
P1234	Pitta	GG	-	-	GA	-	-	-	-	-	-	-	-	-T	-	-	-	-	TG	-	-	TC	-	-
P1252	Kapha	GC	-	-	GA	-	-	-	-	-	-	-	-	-	-	-	-	-	-	-	-	TC	-	-
P442	Kapha	GC	-	-	GA	TT	TT	-	-	-	-	CT	-	-	-	-	TG	-	TG	TC	CA	-	-	-
P542	Kapha	GG	AT	-	-	TT	TT	-	-	-	-	CT	-	-	-	-	TG	-	TG	TC	CA	-	-	-
P728	Kapha	GC	-	-	-	-	-	-	-	-	-	-	-	-	-	-	-	-	-	-	-	-	-	-
P766	Pitta	GG	-	-	-	-	-	-	-	-	-	CT	-	-	-	-	TG	-	TG	TC	CA	-	-	-
P862	Pitta	GC	-	-	-	-	TA	-	-	-	-	-	-	-	-	-	-	-	-	-	-	TC	-	-
P882	Pitta	GG	-	-	-	TC	TA	-	-	-	-	-	-	-	-	-	-	-	-	-	-	-	-	-
P905	Pitta	GC	-	-	-	TC	TA	-	-	-	-	-	-	-	-	-	-	-	TG	-	-	TC	-	-
U040	Kapha	GC	-	-	-	TC	TA	-	-	-	-	CT	-	-	-	AG	TG	-	TG	TC	CA	-	-	-
U181	Pitta	GG	-	-	-	TC	TA	-	-	-	-	-	-	-T	-	-	-	-	-	-	-	TC	-	-
U190	Pitta	GC	-	-	-	-	-	-	-	-	-	-	-	-	-	-	-	-	-	-	-	TC	-	-
U194	Pitta	GC	-	-	-	TC	TA	TG	-	-	TC	-	-	-	-	-	-	-	-	-	-	TC	-	-
U222	Pitta	-	-	-	-	TC	TT	-	-	-	-	CT	-	-	-	-	TG	-	TG	TC	CA	TC	-	-
U223	Pitta	GG	-	-	-	TC	TA	-	-	-	-	-	-	-	-	-	-	-	-	-	-	-	-	-
U226	Pitta	GG	-	-	GA	TC	TA	-	-	-	TC	-	-	-	-	-	-	-	-	-	-	TC	-	-
U238	Pitta	GG	AT	-	-	TC	TT	-	-	-	-	-	-	-	-	-	-	-	-	-	-	-	-	-
U261	Pitta	GG	-	-	-	-	-	-	-	-	-	CT	-	-	-	-	TG	-	TG	TC	CA	-	-	-
U263	Pitta	GG	-	-	-	-	TA	TG	-	-	-	-	-	-	-	-	-	-	TG	-	-	-	-	-
U271	Kapha	GC	-	-	-	-	-	-	-	-	-	CT	-	-	-	-	TG	-	TG	TC	CA	-	-	-
U272	Pitta	GG	-	-	GA	-	TT	-	-	-	TC	-	-	-	-	-	-	-	-	-	-	-	-	-
U288	Pitta	GC	AT	-	-	-	TA	TG	-	-	-	-	-	-	-	-	-	-	-	-	-	-	-	-
U299	Kapha	GC	AT	-	-	TT	TT	-	-	-	-	-	-	-	-	-	-	-	-	-	-	TC	-	-
U300	Kapha	GC	AT	-	-	TC	TA	-	-	-	-	-	-	-	-	-	-	-	-	-	-	TC	-	-
U303	Pitta	GG	-	-	-	TC	TA	-	-	-	-	-	-	-	-	-	-	-	TG	-	-	-	-	-
U315	Pitta	GC	-	-	-	-	-	-	-	-	-	-	-	-	-	-	-	-	-	-	-	TC	-	-

Table S14. The combined association result of Affymetrix and Ion-torrent data.

Platform	Chr	rsID	Physical position	Mutant allele	Frequency of mutant allele in Pitta	Frequency of mutant allele in Kapha	Wild type allele	Chisq value	P-value	OR
Affymetrix	1	rs170091	64053568	T	0.35	0.3724	G	0.1838	0.6681	0.9074
Ion-torrent	1	rs217490	64059644	C	0.2907	0.2571	G	0.2176	0.6409	1.184
Ion-torrent	1	rs61003960	64059863	A	0.1047	0.1429	T	0.5266	0.468	0.7013
Affymetrix	1	rs865929	64064378	T	0.5	0.4586	C	0.5833	0.445	1.18
Affymetrix	1	rs855325	64064780	A	0.125	0.1621	G	0.9087	0.3405	0.7386
Affymetrix	1	rs855319	64068336	A	0.5	0.4552	G	0.685	0.4079	1.197
Affymetrix	1	rs855318	64068386	G	0.5	0.4552	A	0.685	0.4079	1.197
Affymetrix	1	rs855317	64069337	C	0.5083	0.4621	T	0.7284	0.3934	1.204
Affymetrix	1	rs855316	64069495	G	0.1333	0.1724	A	0.9598	0.3272	0.7385
Affymetrix	1	rs855315	64069612	G	0.525	0.4621	A	1.347	0.2459	1.287
Affymetrix	1	rs10889433	64074437	T	0.2167	0.1966	C	0.2127	0.6447	1.131
Affymetrix	1	rs7528129	64076814	C	0.1417	0.1828	T	1.012	0.3144	0.738
Affymetrix	1	rs855300	64078224	A	0.1417	0.169	G	0.4683	0.4938	0.8118
Affymetrix	1	rs11208252	64084491	A	0.1333	0.1966	G	2.318	0.1279	0.6289
Affymetrix	1	rs3790861	64087444	C	0.15	0.1552	T	0.01746	0.8949	0.9608
Ion-torrent	1	Novel	64089405	G	0	0.01429	A	1.236	0.2661	0
Ion-torrent	1	rs855314	64095111	G	0.05814	0.1143	A	1.593	0.207	0.4784
Ion-torrent	1	rs1126728	64097432	T	0.3023	0.4429	C	3.287	0.06984	0.5452
Affymetrix	1	rs2269252	64098065	G	0.275	0.3621	A	2.882	0.08959	0.6683
Affymetrix	1	rs2269251	64098188	A	0.125	0.05862	G	5.197	0.02262	2.294
Ion-torrent	1	rs6588052	64100478	A	0.5233	0.4571	T	0.6748	0.4114	1.303
Ion-torrent	1	Novel	64100693	T	0.03488	0.2	G	10.84	0.0009959	0.1446
Ion-torrent	1	COSM133943	64102058	T	0.01163	0	C	0.8192	0.3654	NA
Affymetrix	1	rs2269248	64102114	C	0.3833	0.3862	T	0.002959	0.9566	0.9879
Ion-torrent	1	rs11577919	64104302	G	0	0.01429	A	1.236	0.2661	0
Ion-torrent	1	rs11581836	64104307	T	0.05814	0.07143	C	0.1136	0.7361	0.8025
Affymetrix	1	rs2284997	64105775	C	0.3833	0.3345	T	0.8918	0.345	1.237
**Affymetrix**	**1**	**rs2269241**	**64108771**	**G**	**0.075**	**0.2621**	**A**	**18.07**	**2.12E-005**	**0.2283**
**Affymetrix**	**1**	**rs2269240**	**64109264**	**G**	**0.075**	**0.2586**	**A**	**17.57**	**2.77E-005**	**0.2324**
**Affymetrix**	**1**	**rs2269239**	**64109359**	**C**	**0.075**	**0.2586**	**G**	**17.57**	**2.77E-005**	**0.2324**
**Affymetrix**	**1**	**rs3790857**	**64111866**	**A**	**0.1667**	**0.3138**	**G**	**9.306**	**0.002284**	**0.4374**
**Affymetrix**	**1**	**rs2024837**	**64112250**	**C**	**0.425**	**0.2931**	**G**	**6.661**	**0.009854**	**1.783**
**Ion-torrent**	**1**	**rs11208257**	**64114301**	**C**	**0.05814**	**0.2**	**T**	**7.261**	**0.007049**	**0.2469**
**Ion-torrent**	**1**	**Novel**	**64114303**	**C**	**0.01163**	**0**	**T**	**0.8192**	**0.3654**	**NA**
**Ion-torrent**	**1**	**Novel**	**64117205**	**A**	**0.03488**	**0**	**T**	**2.49**	**0.1146**	**NA**
**Ion-torrent**	**1**	**rs77434440**	**64117319**	**T**	**0**	**0.01429**	**C**	**1.236**	**0.2661**	**0**
**Ion-torrent**	**1**	**rs147763516**	**64117414**	**A**	**0**	**0.01429**	**G**	**1.236**	**0.2661**	**0**
**Ion-torrent**	**1**	**rs2269238**	**64117537**	**T**	**0.05814**	**0.2**	**G**	**7.261**	**0.007049**	**0.2469**
**Affymetrix**	**1**	**rs2269238**	**64117537**	**A**	**0.075**	**0.2552**	**C**	**17.07**	**3.61E-005**	**0.2367**
Affymetrix	1	rs2269237	64117913	C	0.2417	0.1379	T	6.526	0.01063	1.992
Ion-torrent	1	rs199629577	64120149	T	0	0.01429	C	1.236	0.2661	0
Affymetrix	1	rs2749100	64124979	A	0.1583	0.2621	C	5.131	0.0235	0.5297
Affymetrix	1	rs11208264	64125072	G	0.1667	0.2621	A	4.308	0.03793	0.5632
Ion-torrent	1	rs11208265	64125234	T	0.1628	0.2143	G	0.6762	0.4109	0.713
Affymetrix	1	rs11208265	64125234	T	0.1667	0.2621	G	4.308	0.03793	0.5632
Ion-torrent	1	rs8294	64125368	T	0.05814	0.1857	C	6.153	0.01312	0.2707
Ion-torrent	1	rs4643	64125439	C	0.05814	0.1857	A	6.153	0.01312	0.2707
Affymetrix	1	rs4643	64125439	C	0.08333	0.2276	A	11.7	0.0006262	0.3085
Affymetrix	1	rs2749098	64125476	T	0	0	C	NA	NA	NA
Ion-torrent	1	rs10909	64125544	T	0.186	0.07143	C	4.352	0.03697	2.971
Affymetrix	1	rs10909	64125544	A	0.1583	0.1034	G	2.43	0.119	1.63
Ion-torrent	1	rs80334201	64125793	A	0.01163	0	G	0.8192	0.3654	NA
Ion-torrent	1	Novel	64125944	G	0.01163	0	C	0.8192	0.3654	NA
Affymetrix	1	rs855351	64126450	T	0.15	0.09655	C	2.434	0.1187	1.651
Affymetrix	1	rs855350	64127004	A	0.225	0.3241	G	4.01	0.04523	0.6054
Affymetrix	1	rs855349	64127189	C	0.225	0.3207	A	3.754	0.05268	0.615
Affymetrix	1	rs2749097	64127468	G	0.08333	0.2276	C	11.7	0.0006262	0.3085
Affymetrix	1	rs855346	64127834	A	0.1417	0.08966	G	2.446	0.1178	1.676
Affymetrix	1	rs705510	64127936	G	0.2	0.2793	C	2.802	0.09412	0.6451
Affymetrix	1	rs2013012	64128209	A	0.15	0.2552	G	5.395	0.0202	0.5151

Table S15. Hardy-Weinberg statistical analysis of rs11208257 among different centres used for replication analysis.

Centers	TT	TC	CC	HWE p-value
Bangalore (B)	183	93	9	0.57
Pune (P)	136	70	5	0.368
**Udupi (U)**	**107**	**60**	**2**	**0.04**

Table S16. Association analysis of rs11208257 among 496 *Prakriti* subjects.

Genotypes	Vata	Pitta	Kapha	Total	Vata+Kapha	P-value			
						**P*vs.*V+K**	**P*vs.*V*vs.*K**	**P*vs.*V**	**P*vs.*K**
TT	0.598 (147)	0.759 (88)	0.627 (84)	319	0.608 (231)	0.00616*	0.024*	0.00761*	0.0235*
TC	0.37 (91)	0.207 (24)	0.358 (48)	163	0.366 (139)				
CC	0.033 (8)	0.034 (4)	0.015 (2)	14	0.026 (10)				
Total genotypes	246	116	134	496	380				
T	0.783 (385)	0.862 (200)	0.806 (216)	801	0.791 (601)	0.02061*	0.04031*	0.0149*	0.1203
C	0.217 (107)	**0.138 (32)**	0.194 (52)	191	0.209 (159)				
Total alleles	492	232	268	992	760				

Table S17. Distribution of rs11208257 in Indian and HapMap populations.

Population	TT	TC	CC	Total genotypes	T	C	Total alleles
INDIA	0.674 (747)	0.293 (325)	0.032 (36)	1108	0.821 (1819)	0.179 (397)	2216
ASW	0.623 (33)	0.377 (20)	0 (0)	53	0.811 (86)	0.189 (20)	106
CEU	0.69 (78)	0.31 (35)	0 (0)	113	0.845 (191)	0.155 (35)	226
CHB	0.69 (58)	0.286 (24)	0.024 (2)	84	0.833 (140)	0.167 (28)	168
CHD	0.647 (55)	0.329 (28)	0.024 (2)	85	0.812 (138)	0.188 (32)	170
GIH	0.705 (62)	0.284 (25)	0.011 (1)	88	0.847 (149)	0.153 (27)	176
JPT	0.721 (62)	0.267 (23)	0.012 (1)	86	0.855 (147)	0.145 (25)	172
LWK	0.522 (47)	0.389 (35)	0.089 (8)	90	0.717 (129)	0.283 (51)	180
MEX	0.32 (16)	0.52 (26)	0.16 (8)	50	0.58 (58)	0.42 (42)	100
MKK	0.62 (88)	0.352 (50)	0.028 (4)	142	0.796 (226)	0.204 (58)	284
TSI	0.67 (59)	0.307 (27)	0.023 (2)	88	0.824 (145)	0.176 (31)	176
YRI	0.77 (87)	0.204 (23)	0.027 (3)	113	0.872 (197)	0.128 (29)	226

References

1. Reich, D., Thangaraj, K., Patterson, N., Price, A. L., Singh, L. Reconstructing Indian population history. *Nature* **461**, 489-494 (2009).

2. Moorjani, P., *et al.* Genetic evidence for recent population mixture in India. *Am. J. Hum.Genet.* **93**, 422-438 (2013).
